# Gapmers targeting conserved SARS-CoV-2 single-stranded regions reduce viral load and reshape inflammatory macrophage responses

**DOI:** 10.1016/j.isci.2026.117639

**Published:** 2026-09-28

**Authors:** Greta Gallo, Lorena Donnici, Liam S. Colley, Alessia Loffreda, Valeria Fumagalli, Matteo Iannacone, Davide Mazza, Danny Incarnato, Raffaele De Francesco, Emanuele Monteleone, Alessio De Magis, Marco E. Bianchi

**Affiliations:** 1 School of Medicine, Vita-Salute San Raffaele University, Milan, Italy; 2 Istituto Nazionale Genetica Molecolare “Romeo ed Enrica Invernizzi”, Milan, Italy; 3 HMGBiotech, Milan, Italy; 4 Experimental Imaging Center, IRCCS Ospedale San Raffaele, Milan, Italy; 5 Division of Immunology, Transplantation and Infectious Diseases, IRCCS Ospedale San Raffaele, Milan, Italy; 6 Department of Molecular Genetics, Groningen Biomolecular Sciences and Biotechnology Institute (GBB), University of Groningen, Groningen, the Netherlands; 7 Department of Pharmacological and Biomolecular Sciences (DiSFeB), University of Milan, Milan, Italy; 8 Chromatin Dynamics Unit, Division of Genetics and Cell Biology, IRCCS Ospedale San Raffaele, Milan, Italy

**Keywords:** nucleic acid therapeutics, antisense oligonucleotides, SARS-CoV-2, antiviral response, RNA-targeted therapy

## Abstract

The COVID-19 crisis is now over, but additional viral strains of SARS-CoV-2 that evade immunity are continuously emerging. We explored the design, evaluation, and optimization of anti-sense oligonucleotides (ASOs) targeting SARS-CoV-2 RNA, with the aim of providing a scalable strategy capable of responding to emergent outbreaks. Twenty-four ASOs were designed to target highly conserved, single-stranded regions of the SARS-CoV-2 genome. Extensive *in vitro* screening led to the identification of gapmers that exhibited high efficacy against four selected SARS-CoV-2 variants, significantly reducing intracellular viral RNA and virus production. Importantly, the antiviral efficacy correlates with viral RNA accessibility and is not due to activation of antiviral host responses. Finally, naked gapmers delivered intranasally can reach lung tissue, are preferentially internalized by alveolar macrophages, and modulate the inflammatory response to viral exposure. Taken together, our findings show that gapmers targeting the viral RNA are a promising approach to the development of antiviral therapies.

## Highlights

• Gapmers targeting conserved SARS-CoV-2 ssRNA regions reduce viral load
• Gapmer efficacy correlates with RNA structural accessibility (SHAPE-MaP)
• Antiviral effect stems from RNA cleavage, not interferon activation
• Intranasal gapmers reach lung macrophages and reshape inflammation

## Graphical abstract

**Figure:**
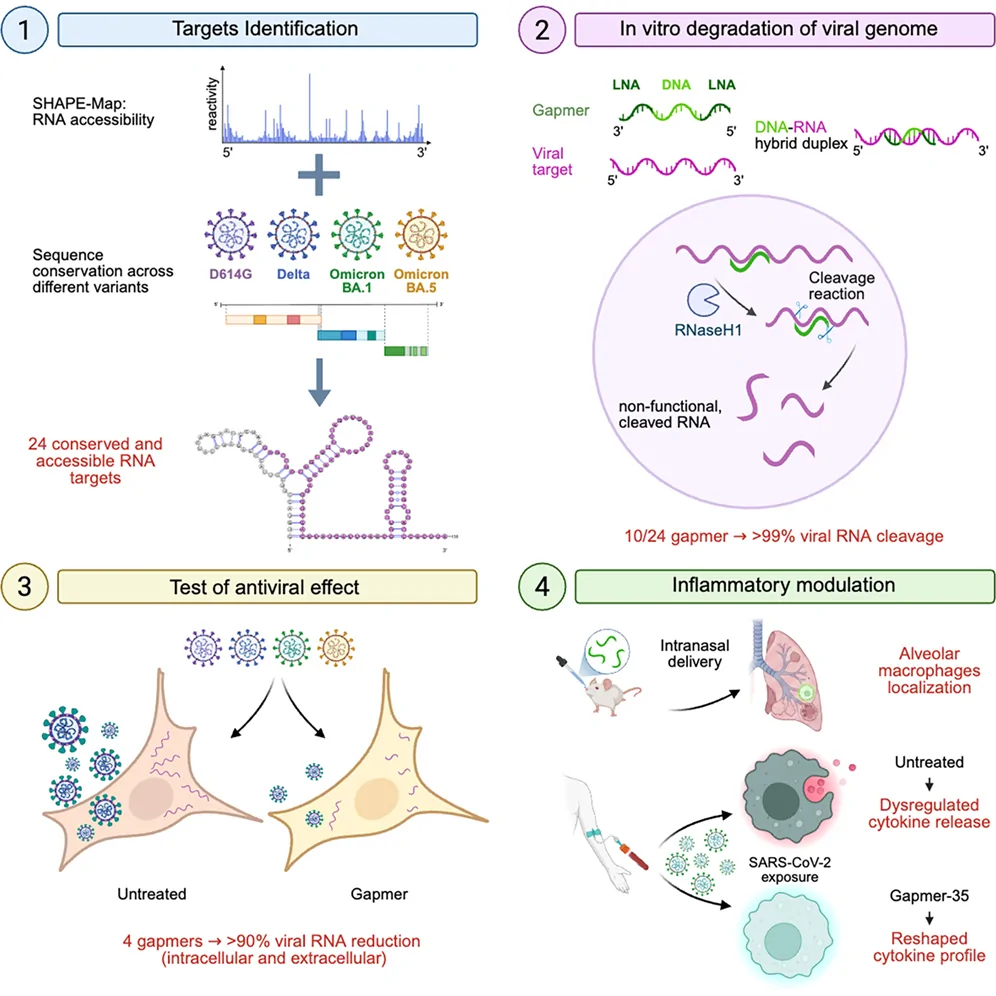

## Teaser

Biological sciences; Microbiology; Virology

## Introduction

*Coronaviridae* gained global recognition due to multiple serious outbreaks, including the Coronavirus disease 2019 (COVID-19) pandemic. Research on coronaviruses dates back to the early 1930s.^1^ The prominence of coronaviruses surged in 2003 with the emergence of the severe acute respiratory syndrome coronavirus (SARS-CoV) epidemic, and in 2012 when Middle East respiratory syndrome coronavirus (MERS-CoV) triggered a second epidemic.^2^ In the absence of specific antivirals or vaccines, both epidemics were ultimately controlled through conventional methods, such as isolating patients and restricting travel.^2^^,^^3^ SARS-CoV-2, the virus responsible for COVID-19, is the seventh known coronavirus to affect humans^4^ and the third to result in fatal infections.^5^ SARS-CoV-2 originated in bats and was first identified in Wuhan, Hubei province, China.^6^ In 2019, SARS-CoV-2 was declared a pandemic. In 2022, the World Health Organization (WHO) reported over 110 million confirmed COVID-19 cases and nearly 2.5 million deaths worldwide (World Health Organization website). SARS-CoV-2 spreads much more rapidly than previous coronaviruses (SARS-CoV and MERS-CoV) due to transmission during the early, asymptomatic stage of infection.^5^^,^^6^

The SARS-CoV-2 genome has ≈80% identity with SARS-like coronavirus.^7^ It is characterized by a 5′ capped and 3′ polyadenylated single stranded, linear, positive sense RNA (ssRNA+).^8^ The genome contains 5′ and 3′ untranslated regions and *cis*-acting secondary RNA structures,^8^^,^^9^ and encodes 29 structural and non-structural proteins, including open reading frame 1a/b (ORF1a/b) polyproteins, spike (S), envelope (E), membrane (M), nucleocapsid (N1), and accessory proteins.^8^^,^^9^

SARS-CoV-2 infection requires the binding of the viral S protein with a host cell receptor, Angiotensin-converting enzyme 2 (ACE2).^10^ Then, the S protein undergoes conformational changes that facilitate membrane fusion, allowing the viral genome to enter the host.^10^ The cleavage of the S protein by host proteases, such as Transmembrane protease serine 2 (TMPRSS2), is crucial for this process as it activates the fusion machinery of the virus.^11^ Modulation of TMPRSS2 expression increased or decreased the sensibility to viral infections.^11^^,^^12^ However, beyond the productive infection of the respiratory epithelium through this canonical route, the virus is concurrently detected by the lung’s resident immune compartment, thereby initiating a localized immune surveillance response.^13^

Alveolar macrophages (AMs) serve as the primary sentinels in the lung, rapidly internalizing SARS-CoV-2 particles upon exposure.^14^ Clinical evidence from bronchoalveolar lavage fluid (BALF) of COVID-19 patients frequently reveals viral RNA and protein within these cells, a phenomenon indicative of restrictive uptake rather than productive replication.^14^^,^^15^ While this internalization is a fundamental component of the host’s innate defense, in the context of SARS-CoV-2 it often functions as a critical trigger for a dysregulated, hyper-inflammatory trajectory. This initial sensing event catalyzes a self-amplifying secretory program, called the ‘cytokine storm', which drives the severe tissue pathology and morbidity associated with the disease.^15^^,^^16^

This secretory program in the macrophage response to SARS-CoV-2 involves a coordinated set of pro- and anti-inflammatory cytokines that drive downstream type I interferon signaling,^17^^,^^18^ antiviral interferons (IFN-β, IFN-γ), interferon-stimulated chemokines like C-X-C motif chemokine ligand 10 (CXCL10), core inflammatory mediators like Tumor Necrosis Factor (TNF-α) and interleukins (IL-1β, IL-6, IL-8, IL-12), and regulators of myeloid function such as Granulocyte-macrophage colony-stimulating factor (GM-CSF), alongside Interleukin-1 Receptor Antagonist (IL-1ra), which provides anti-inflammatory feedback.^19^^,^^20^^,^^21^^,^^22^ Infected macrophages do not transition into classical lytic cell death but instead enter a state of ‘abortive pyroptosis’, characterized by a persistent inflammatory program that produces a robust yet pathological output of cytokines, such as IL-6 and CXCL10, which fuel the intense pulmonary inflammation associated with severe COVID-19.^17^^,^^23^

Further to its intrinsic pathogenicity, SARS-CoV-2 undergoes rapid evolution and diversification,^24^ leading to the emergence of more infectious variants. To date, the WHO has categorized as potential health risks the viral variants D614G, Delta, Omicron BA.1 and BA.5. The D614G mutation involves a single amino acid substitution in the S protein of SARS-CoV-2, where aspartic acid (D) at position 614 is replaced by a glycine (G); the D416G variant quickly supplanted the original Wuhan strain, becoming the predominant form of the virus by mid-2020.^25^ The Delta variant was first identified in October 2020 and soon became the predominant variant due to mutations that facilitate viral entry into host cells.^26^^,^^27^ The Omicron variant BA.1, first identified in late 2021, is characterized by a large number of mutations associated with altered transmissibility and pathogenicity compared to earlier variants. Notably, Omicron variant BA.5 has mutations, especially in the S protein, that increase its ability to escape immune responses^28^ and thus decrease the effectiveness of commercial vaccines.

The higher mutation rate of the later variants and the reduced effectiveness of vaccines in immunocompromised patients (who are among those most at risk of death after infection) have pushed researchers to seek alternative solutions. An emerging class of therapeutic agents that have garnered significant attention in recent years for their potential to reduce viral load are Antisense Oligonucleotides (ASOs). ASOs can be designed to bind specific viral RNA sequences (e.g., S protein mRNA) and prevent their translation or trigger degradation. ASOs do not rely on immune activation, so they could be used in vulnerable patients such as immunocompromised patients.

The therapeutic efficacy of ASOs is significantly enhanced through various chemical modifications that improve their stability, affinity, and overall pharmacological properties such as phosphorothioate (PS) and Locked Nucleic Acid (LNA) modifications. The former replaces one of the non-bridging oxygen atoms in the phosphodiester group with sulfur; the latter involves the incorporation of a methylene bridge that connects the 2′ oxygen and the 4′ carbon of the ribose sugar, effectively “locking” the nucleic acid in a rigid conformation. Both modifications lead to increased thermal stability and resistance to nucleases and improve the binding affinity of ASOs to their target RNA, facilitating effective gene silencing.^29^^,^^30^^,^^31^ ASOs that contain modifications only at their 5′ and 3′ ends are called gapmers. Gapmers have shown significant potential in various viral infections, including hepatitis C and SARS-CoV-2, by effectively reducing viral loads and inhibiting replication.^32^^,^^33^^,^^34^

Despite the promising results obtained by several gapmers against SARS-CoV-2 in reducing viral loads in a mouse model,^35^ a clear methodological framework for their design is still missing. Thus, in this work we developed an in-silico workflow incorporating RNA structural data and conservation metrics, providing a robust rationale for gapmer design. We then showed that a high proportion of these gapmers significantly reduce viral RNA levels across multiple SARS-CoV-2 variants. Furthermore, we showed that in infected macrophages gapmers can resolve the persistent dysregulated inflammatory state and promote the transition to a more regulated and physiological secretory profile.^36^^,^^37^ Ultimately, our study highlights the promise of RNA-based therapeutics against *Coronaviridae* or other ssRNA viruses, particularly for vulnerable populations such as immunocompromised individuals.

## Results

### Identification of SARS-CoV-2 genomic regions that are both single-stranded and conserved

Our strategy for ASO design was based on the structural organization of the SARS-CoV-2 RNA genome, with the aim of identifying structurally accessible target regions that were also evolutionarily conserved. Overall, target prioritization was based on two criteria (Figure 1A): (i) conservation of structural accessibility, and (ii) sequence conservation across SARS-CoV-2 VoCs and SARS-CoV (Figure 1A).

**Figure 1:**
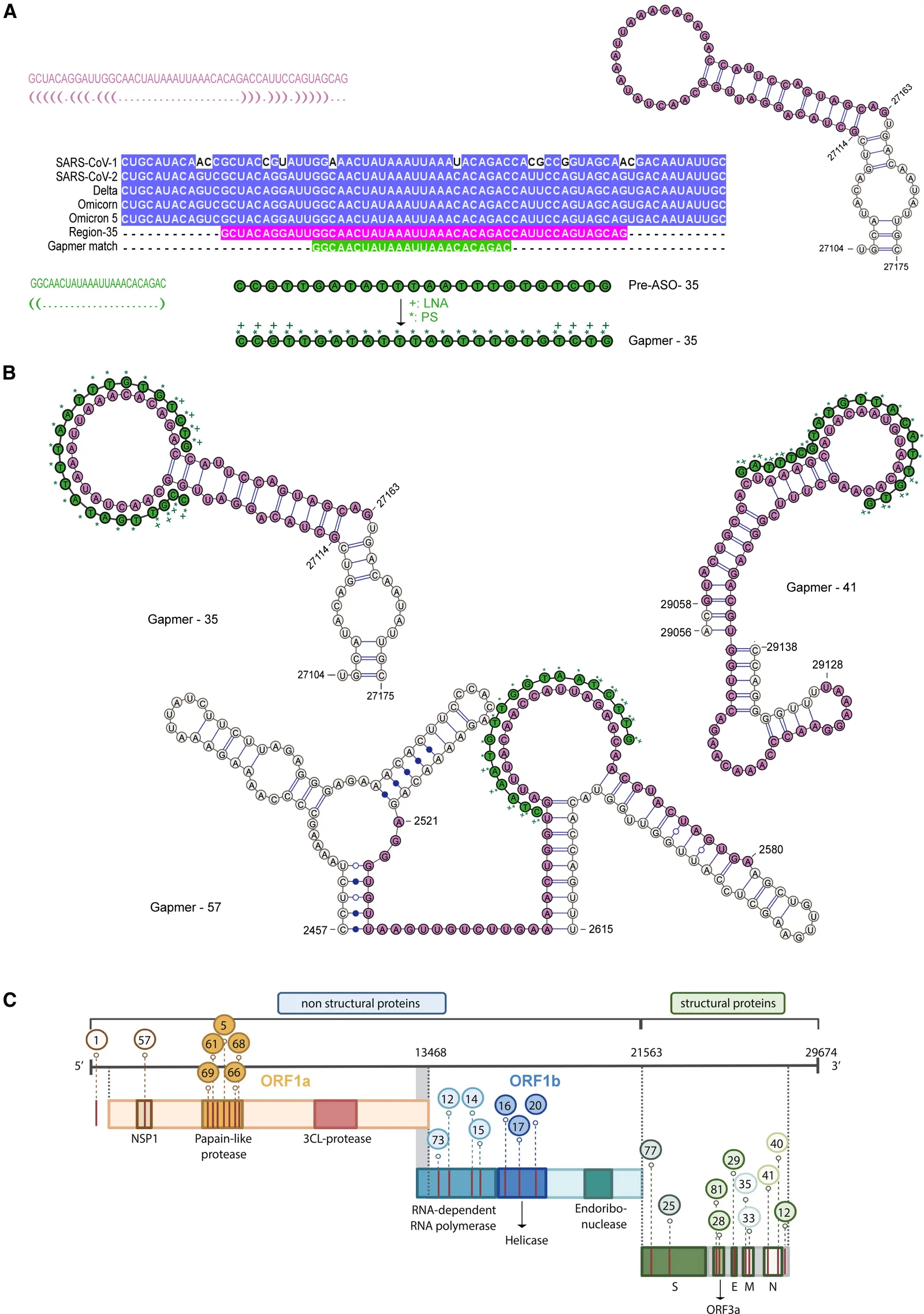
Design of selected SARS-CoV-2 target regions (A) Schematic representation of the gapmer design pipeline, using Gapmer-35 as an example. Structurally accessible regions within the SARS-CoV-2 genome identified by Manfredonia et al.^38^ are highlighted in magenta and shown as nucleotide sequence and corresponding dot-bracket notation (top left), as well as in the predicted folded RNA structure (top right). Sequence conservation of the selected region was evaluated by multiple sequence alignment of SARS-CoV-2, SARS-CoV and the VoCs (middle). Conserved nucleotides are shown in blue, whereas non-conserved positions are shown in white. The selected target region is highlighted in magenta and the corresponding gapmer target sequence is highlighted in green. The antisense sequence complementary to the selected target was initially designed as a pre-ASO and subsequently converted into a gapmer through the introduction of phosphorothioate (PS) linkages throughout the oligonucleotide backbone and LNA modifications at both termini (bottom). “+” indicates LNA-modified nucleotides and “∗” indicates phosphorothioate linkages. (B) Structural accessibility of the selected target regions and their localization within the surrounding RNA architecture. Structures were generated using the VARNA software based on the secondary structure model of SARS-CoV-2.^38^ Coordinates refer to the SARS-CoV-2 reference genome Leiden-0002. Exemplary target sequences are highlighted in magenta, whereas the corresponding gapmers are in green. Within the gapmer sequences, the symbol “+” denotes LNA-modified nucleotides, whereas “∗” indicates phosphorothioate linkages. See also Figures S1–S4. (C) Schematic representation of the SARS-CoV-2 genome highlighting key protein-coding regions, including the RNA-dependent RNA polymerase, protease, and structural proteins. Numbers in circles indicate the positions of 24 selected gapmers designed against specific genomic regions. Image created using BioRender.

First, candidate regions were selected based on the genome-wide RNA structural analysis previously reported,^38^ where SARS-CoV-2 RNA secondary structure was experimentally mapped using the primer extension and mutational profiling (SHAPE-MaP) (Figure 1A). Candidate sequences were then checked for complete sequence conservation among VoCs using Clustal Omega implemented in Jalview (Figure S1). Almost all sequences passed this filter (column F, Table 1), and were then stratified according to sequence conservation relative to SARS-CoV (Column G, Table 1), including 16 regions displaying conservation scores >0.8 between SARS-CoV-2 and SARS-CoV and 8 additional regions with conservation scores >0.6 (Table 2). To further refine our target selection, dot-bracket secondary structure annotations were analyzed using custom R scripts. The first script “extractDot” (Data S1) extracted the dot-bracket segment corresponding to each candidate region and identified consecutive runs of dots, representing unpaired nucleotides. For each candidate region, the longest continuous unpaired stretch was selected as the most structurally accessible target site. The corresponding nucleotide sequence was then processed using a second script (“revComp”), which generated the reverse-complement antisense sequence directed against the selected target (Data S1). Using this pipeline, we identified 24 ASO sequences ranging from 18 to 22 nucleotides in length (Figures 1A, 1B, and S2–S4).

**Table 1:** Selection criteria and sequence conservation of candidate gapmer target regions – related to Figure 1

Sequence Name	From -	To (# nt)	Sequence target	length (nt)	VoC conserved	SARS-CoV conservation
						>0,8
1	134	161	AATAACTAATTACTGTCGTTGACAGGA	27	YES	0,84788
2	289	331	AACGAGAAAACACACGTCCAACTCAGTTTGCCTGTTTTACAG	42	YES	0,92736
3	627	669	TCTTCTTCGTAAGAACGGTAATAAAGGAGCTGGTGGCCATAG	42	YES	0,90391
4	809	851	ACACTCGCTATGTCGATAACAACTTCTGTGGCCCTGATGGCT	42	YES	0,88037
5	3344	3389	GTTTTAGTGGTTATTTAAAACTTACTGACAATGTATACATTAAAA	45	YES	0,83664
6	4471	4498	CAGCGTAAATATAAGGGTATTAAAATA	27	YES	0,81511
7	5156	5181	CTTTTGAGTACTACCACACAACTGA	25	YES	0,81133
8	8749	8786	AACAAACATGCTGATTTTGACACATGGTTTAGCCAGC	37	YES	0,91672
9	10698	10724	AGACAGGTGGTTTCTCAATCGATTTA	26	YES	0,80826
10	11606	11676	TAGTTTATTGTTTCTTAGGCTATTTTTGTACTTGTTACTTTGGCCTCTT TTGTTTACTCAACCGCTACTT	70	YES	0,84559
11	12956	12990	TTAAAGGATTAAACAACCTAAATAGAGGTATGGT	34	YES	0,90606
12	13452	13481	ACAATCGTTTTTAAACGGGTTTGCGGTGT	29	YES	0,89191
13	13662	13744	CACACTTTCTCTAACTACCAACATGAAGAAACAATTTATAATTTACTTAAG GATTGTCCAGCTGTTGCTAAACATGACTTCT	82	YES	0,82453
14	15782	15865	CTTTAAGTCAGTTCTTTATTATCAAAACAATGTTTTTATGTCTGAAGCAAAA TGTTGGACTGAGACTGACCTTACTAAAGGAC	83	YES	0,93509
15	15955	16048	GGGCCGGCTGTTTTGTAGATGATATCGTAAAAACAGATGGTACACTTAT GATTGAACGGTTCGTGTCTTTAGCTATAGATGCTTACCCACTTA	93	YES	0,88578
16	16492	16543	ATGGACAAGTTTTTGGTTTATATAAAAATACATGTGTTGGTAGCGATAATG	51	YES	0,84520
17	16770	16820	AATTATGTCTTTACTGGTTATCGTGTAACTAAAAACAGTAAAGTACAAAT	50	YES	0,89881
18	17281	17311	ATTCAACATTAGAACAGTATGTCTTTTGTA	30	YES	0,86977
19	17477	17515	GGGCACACTAGAACCAGAATATTTCAATTCAGTGTGTA	38	YES	0,91991
20	17638	17665	CAGCTCAATGCTTTAAAATGTTTTATA	27	YES	0,89183
21	18346	18396	CCAATTTACCTTTACAGCTAGGTTTTTCTACAGGTGTTAACCTAGTTGCT	50	YES	0,83733
22	18722	18766	TCATTCTATTGGATTTGATTACGTCTATAATCCGTTTATGATTG	44	YES	0,83868
23	21050	21090	GTACGACCCTAAGACTAAAAATGTTACAAAAGAAAATGAC	40	YES	0,84934
24	21385	21411	CTTTATTTGACATGAGTAAATTTCCC	26	YES	0,81847
25	23548	23582	GACATACCCATTGGTGCAGGTATATGCGCTAGTT	34	YES	0,80024
26	24267	24300	GGCTTATAGGTTTAATGGTATTGGAGTTACACA	33	YES	0,85548
27	25534	25611	TTGGCGTTGCACTTCTTGCTGTTTTTCAGAGCGCTTCCAAAATCATAACC CTCAAAAAGAGATGGCAACTAGCACTC	77	YES	0,84462
28	25781	25852	CTGGAAATGCCGTTCCAAAAACCCATTACTTTATGATGCCAACTATTTTC TTTGCTGGCATACTAATTGTT	71	YES	0,80453
29	26369	26400	CTGCTGCAATATTGTTAACGTGAGTCTTGTA	31	YES	0,93480
30	26411	26436	TTACGTTTACTCTCGTGTTAAAAAT	25	YES	0,92450
31	26588	26635	GTAATAGGTTTCCTATTCCTTACATGGATTTGTCTTCTACAATTTGC	47	YES	0,82425
32	26655	26687	TTTTGTATATAATTAAGTTAATTTTCCTCTGG	32	YES	0,84437
33	26847	26900	TGTGGTCATTCAATCCAGAAACTAACATTCTTCTCAACGTGCCACTCCATGGC	53	YES	0,86305
34	26992	27026	ACGCTGTGACATCAAGGACCTGCCTAAAGAAATC	34	YES	0,88101
35	27114	27163	GCTACAGGATTGGCAACTATAAATTAAACACAGACCATTCCAGTAGCAG	49	YES	0,81276
36	27484	27532	TAAAAGAACCTTGCTCTTCTGGAACATACGAGGGCAATTCACCATTTC	48	YES	0,93088
37	28261	28290	GAACAAACTAAAATGTCTGATAATGGACC	29	NO	0,96337
38	28400	28439	AAGGTTTACCCAATAATACTGCGTCTTGGTTCACCGCTC	39	YES	0,96893
39	28512	28560	AGATGACCAAATTGGCTACTACCGAAGAGCTACCAGACGAATTCGTGG	48	YES	0,95557
40	28669	28722	GTTGCAACTGAGGGAGCCTTGAATACACCAAAAGATCACATTGGCACCCGCAA	53	YES	0,95466
41	29058	29128	TACTGCCACTAAAGCATACAATGTAACACAAGCTTTCGGCAGACGTGGT CCAGAACAAACCCAAGGAAAT	70	YES	0,85104
42	29288	29412	TGGATGACAAAGATCCAAATTTCAAAGATCAAGTCATTTTGCTGAATAAGC ATATTGACGCATACAAAACATTCCCACCAACAGAGCCTAAAAAGGACAAA AAGAAGAAGGCTGATGAAACTCA	124	NO	0,89307
43	29474	29502	ATGATTTCTCCAAACAATTGCAACAATC	28	YES	0,82716
44	29610	29670	GTGCAGAATGAATTCTCGTAACTACATAGCACAAGTAGATGTAGTTAACTT TAATCTCAC	60	YES	0,92970
45	29726	29806	TTTCACCGAGGCCACGCGGAGTACGATCGAGTGTACAGTGAACAATGCT AGGGAGAGCTGCCTATATGGAAGAGCCCTAA	80	NO	0,94178

						**<0,8**

50	23	85	GTAACAAACCAACCAACTTTCGATCTCTTGTAGATCTGTTCTCTAAACGAA CTTTAAAATCT	62	NO	0,79986
51	495	566	AACTGCACCTCATGGTCATGTTATGGTTGAGCTGGTAGCAGAACTCGAA GGCATTCAGTACGGTCGTAGTG	71	YES	0,73022
52	871	941	GACCTTCTAGCACGTGCTGGTAAAGCTTCATGCACTTTGTCCGAACAAC TGGACTTTATTGACACTAAGA	70	YES	0,69043
53	1467	1499	TCGCACTATTGCCTTTGGAGGCTGTGTGTTCT	32	YES	0,72403
54	1719	1813	TGCTTTTGTGGAAACTGTGAAAGGTTTGGATTATAAAGCATTCAAACAAA TTGTTGAATCCTGTGGTAATTTTAAAGTTACAAAAGGAAAAGCT	94	YES	0,71436
55	2199	2229	TTGGGAAATTGTTAAATTTATCTCAACCTG	30	YES	0,70822
56	2252	2326	AAATTGTCACCTGTGCAAAGGAAATTAAGGAGAGTGTTCAGACATTCTTT AAGCTTGTAAATAAATTTTTGGCT	74	YES	0,49898
57	2521	2580	ACAGAGGAAGTTGTCTTGAAAACTGGTGATTTACAACCATTAGAACAACC TACTAGTGA	59	YES	0,60425
58	2626	2650	CTTATGTTGCTCGAAATCAAAGAC	24	YES	0,67638
59	3400	3426	GTGGAAGAAGCTAAAAAGGTAAAACC	26	YES	0,56912
60	3693	3717	TCTACTTGCACCATTATTATCAGC	24	YES	0,79753
61	4808	4847	CCTATTCTGGACAATCTACACAACTAGGTATAGAATTTC	39	YES	0,77263
62	5064	5106	TGGAGCTGATGTTACTAAAATAAAACCTCATAATTCACATGA	42	YES	0,76342
63	5460	5488	GAGTTACTTGTTTCAACATGCCAATTTA	28	YES	0,62920
64	5613	5662	TCAGATACCTTGTACGTGTGGTAAACAAGCTACAAAATATCTAGTACAA	49	NO	0,73416
65	5780	5811	TAACTTCTAAAGAAACTTTGTATTGCATAGA	31	YES	0,72004
66	5864	5901	TCTACAAAGAAAACAGTTACACAACAACCATAAAACC	37	YES	0,75460
67	6240	6275	GCATGTTAACAATGCAACTAATAAAGCCACGTATA	35	YES	0,63706
68	6829	6857	AGAATTAAAGCATCTATGCCGACTACTA	28	YES	0,75663
69	6874	6941	AAGAGTGTCGGTAAATTTTGTCTAGAGGCTTCATTTAATTATTTGAAGTCA CCTAATTTTTCTAAAC	67	YES	0,77439
70	7131	7156	TGTTTGTCTTAGTGGTTTAGATTCT	25	YES	0,71035
71	10362	10393	TGTTCGCATTCAACCAGGACAGACTTTTTCA	31	YES	0,78768
72	11544	11573	TATGTGTGTTGAGTATTGCCCTATTTTCT	29	YES	0,79835
73	14153	14194	TTCATTGTTAATGCCTATATTAACCTTGACCAGGGCTTTAA	41	YES	0,70694
74	22068	22124	ATATGTCTCTCAGCCTTTTCTTATGGACCTTGAAGGAAAACAGGGTAATTTCAAAA	56	YES	0,56639
75	22140	22164	GTTTAAGAATATTGATGGTTATTT	24	YES	0,71671
76	23292	23320	TGATCCACAGACACTTGAGATTCTTGAC	28	YES	0,66780
77	23422	23500	CCTGTTGCTATTCATGCAGATCAACTTACTCCTACTTGGCGTGTTTATTCT ACAGGTTCTAATGTTTTTCAAACACGT	78	YES	0,70829
78	24317	24352	ACCAAAAATTGATTGCCAACCAATTTAATAGTGCT	35	YES	0,77846
79	25388	25437	ACTTATGGATTTGTTTATGAGAATCTTCACAATTGGAACTGTAACTTTG	49	YES	0,73564
80	25635	25660	AACTTGCTGTTGTTGTTTGTAACAG	25	YES	0,69658
81	25716	25741	TATGCTTTAGTCTACTTCTTGCAGA	25	YES	0,61649
82	27242	27320	ATTACTAATTATTATGAGGACTTTTAAAGTTTCCATTTGGAATCTTGATTAC ATCATAAACCTCATAATTAAAAATTT	78	NO	0,68360
83	27814	27895	TGTTTTAATTATGCTTATTATCTTTTGGTTCTCACTTGAACTGCAAGATCATAATGAAACTTGTCACG CCTAAACGAACAT	81	NO	0,79665
84	28046	28070	AGAAAATCAGCACCTTTAATTGAA	24	YES	0,21070

Columns report the region name (A), genomic coordinates (B-C), target sequence (D), target length in nucleotides (nt) (E), sequence conservation across SARS-CoV-2 VoCs (Yes/No) (F), and sequence conservation score between SARS-CoV-2 and SARS-CoV (>0.8 and <0.8) (G). Sequence conservation across SARS-CoV-2 VoCs and between SARS-CoV-2 and SARS-CoV were used as selection criteria for target prioritization.

**Table 2:** Sequence and structural features of the selected ASO targets – related to Figure 1

Sequence Name	SARS-CoV conservation	ASO Name	ASO sequence	Dot-brackets target	Protein match
1	>0.8	ASO 1	GTCAACGACAGTAATTAG	....**...........((((((((**(((.	UTR
5	>0.8	ASO 5	GTCAGTAAGTTTTAAATAAC	)))).))))**)))...............((**(((((...(((.....	PL-PRO
12	>0.8	ASO 12	CCGCAAACCCGTTTAAAAACG	)....**...........))))))))))**...	POL
14	>0.8	ASO 14	CATTGTTTTGATAATAAAG	((.......))))**)))))..............**....(((((.(((((((((((.......((((.....(((......)))..	POL
15	>0.8	ASO 15	GAACCGTTCAATCATAAGTGTAC	))...))(((((((...............)))))))..)**))))))............(((..**((((((........))))))..)))......	POL
16	>0.8	ASO 16	CACATGTATTTTTATATAAACC	)))).........)**))))))...............(**((((((((......)	HEL
17	>0.8	ASO 17	GTTTTTAGTTACACGATAACC	.....((((((((((**(...................)**))))))).)))...	HEL
20	>0.8	ASO 20	CATTTTAAAGCATTGAGCTG	**)))).)))))))........**.......	HEL
25	>0.8	ASO 25	CTAGCGCATATACCTGCACCAATG	)))))...**.....)))))).............**..	S1
28	>0.8	ASO 28	GGCATCATAAAGTAATGGG	)......))).)))))))...**..............(((((**(..............)))))).......(((	ORF3A
29	>0.8	ASO 29	CGTTAACAATATTGCAGCAG	**.................(((**.((((...(((	ENV
33	>0.8	ASO 33	GAGAAGAATGTTAGTTTCTGG	)..))).)))))))**.....................**...((((((.....))))	MEMB
35	>0.8	ASO 35	GTCTGTGTTTAATTTATAGTTGCC	(((((.(((.(**((.....................)**)).))).)))))..	MEMB
40	>0.8	ASO 40	GTGATCTTTTGGTGTATTC	(((((......(((.(((..**...................**...))).)))))))	NP
41	>0.8	ASO 41	GTGTTACATTGTATGCTTTAG	(.(((((.**..(((((..............**..))))).)))))))))(((((.........((((......	NP
44	>0.6	ASO 44	GTTACGAGAATTCATTCTGCAC	**)))...............((((**(((((...........))))))))).............	ORF10
57	>0.6	ASO 57	GTTCTAATGGTTGTAAATC	..)))))............((((((((**(..................**.(((.(((((((.	NSP2
61	>0.6	ASO 61	GAAATTCTATACCTAGTTGTG	((......))))))))).**...................((**	PL-PRO
66	>0.6	ASO 66	GTTTTATGGTTGTTGTGTAACTG	(((....))))))**))....................**((	PL-PRO
68	>0.6	ASO 68	GGCATAGATGCTTTAATTC	.**...............((((**(((.(((.	PL-PRO
69	>0.6	ASO 69	GAAGCCTCTAGACAAAATTTAC	)..)))))))**))).............((((((**(..........))))))).................	PL-PRO
73	>0.6	ASO 73	CCCTGGTCAAGGTTAATATAG	))....(((((.((**((................)))**).))))	POL
77	>0.6	ASO 77	GTAGGAGTAAGTTGATCTGC	((((.(((.......**)))................(**(((((((((((.....(((........))).....))))))))	S1
81	>0.6	ASO 81	CAAGAAGTAGACTAAAGC	...**............((((((**((((	ORF3A

UTR, Untranslated Region; POL, Polymerase; HEL, Helicase; S1, Spike Subunit 1; ORF, Open Reading Frame; ENV, Envelope; MEMB, Membrane; NP, Nucleoprotein; NSP, Non-Structural Protein; PL-PRO, Papain-Like Protease.

Bold: actual target of Gapmers

Columns report for each selected region the region name (A), the conservation score (B), the corresponding ASO name (C), ASO sequence (D), dot-bracket notation of the corresponding SARS-CoV-2 target region (E), with the nucleotides complementary to the ASO highlighted in **bold**, and the viral protein-coding region containing the target sequence (F).

Overall, the selected target regions have remained remarkably conserved throughout the course of the pandemic, suggesting that they are subject to strong evolutionary constraints and may therefore retain therapeutic relevance for future SARS-CoV-2 variants.^39^ Notably, many of these sequences correspond to protein coding regions, such as polymerase, protease, and structural proteins, as illustrated in Figure 1C. This is expected, since protein-coding regions are generally subject to more severe selection pressure.

### Gapmers targeting highly conserved SARS-CoV-2 genomic regions effectively reduce viral load *in vitro* and in cells

The reverse complement of the 24 sequences initially selected were chemically synthesized as unmodified antisense DNA oligonucleotides, generating what we call preliminary antisense oligonucleotides, or pre-ASOs (Figure 1A). Then, we evaluated *in vitro* whether the pre-ASOs could facilitate RNase H cleavage of the viral RNA (Figure S5A). RNase H specifically cleaves RNA/DNA hybrids; therefore, the amount of viral RNA remaining after RNAse H digestion is inversely proportional to the efficiency of RNase H-mediated cleavage. We reverse-transcribed (RT) the viral RNA incubated with RNAse H with 2 pre-ASO concentrations, 5 and 10 μM, and performed qPCR spanning the sequences matching the pre-ASOs. In this assay, ten pre-ASOs achieved at least 99% cleavage (Figure S5A).

We then introduced locked nucleic acid (LNA) modifications to these ten pre-ASOs to enhance their bioavailability and stability in a cellular system (Table 3). Since ASOs where all nucleotides are LNA do bind complementary RNA with high affinity, but are not good targets for RNAseH, we designed ASOs where LNA is present only at the 5′ and 3′ ends. Moreover, all phosphates in the ASO backbone were modified to phosphorothioates (PS); for the sake of simplicity, we will call these antisense oligonucleotides “gapmers”(Figures 1A, 1B, and S2–S4). Gapmers were previously used by others, with excellent results.^35^

**Table 3:** Gapmer design and target sequences – related to Figure 1

Sequence Name	Gapmer Name	Gapmer	Sequence target
15	gapmer 15	+G*+T*+T*+C*T*A*A*T*G*G*T*T*G*T*A*+A*+A*+T*+C	GGGCCGGCTGTTTTGTAGATGATATCGTAAAAACAGATG **GTACACTTATGATTGAACGGTTC**GTGTCTTTAGCTATAG ATGCTTACCCACTTA
25	gapmer 25	+C*+T*+A*+G*+C*G*C*A*T*A*T*A*C*C*T*G*C*A*C*+C*+A*+A*+T*+G	GACATACC**CATTGGTGCAGGTATATGCGCTAG**TT
28	gapmer 28	+G*+G*+C*+A*T*C*A*T*A*A*A*G*T*A*A*+T*+G*+G*+G	CTGGAAATGCCGTTCCAAAAA**CCCATTACTTTATGATGCC** AACTATTTTCTTTGCTGGCATACTAATTGTT
33	gapmer 33	+G*+A*+G*+A*A*G*A*A*T*G*T*T*A*G*T*T*T*+C*+T*+G*+G	TGTGGTCATTCAAT**CCAGAAACTAACATTCTTCTC** AACGTGCCACTCCATGGC
35	gapmer 35	+G*+T*+C*+T*G*T*G*T*T*T*A*A*T*T*T*A*T*A*G*T*+T*+G*+C*+C	GCTACAGGATT**GGCAACTATAAATTAAACACAG** ACCATTCCAGTAGCAG
40	gapmer 40	+G*+T*+G*+A*T*C*T*T*T*T*G*G*T*G*T*+A*+T*+T*+C	GTTGCAACTGAGGGAGCCTT**GAATACACCAAAAGATCAC** ATTGGCACCCGCAA
41	gapmer 41	+G*+T*+G*+T*T*A*C*A*T*T*G*T*A*T*G*C*T*+T*+T*+A*+G	TACTGCCA**CTAAAGCATACAATGTAACAC**AAGCTTTCGGC AGACGTGGTCCAGAACAAACCCAAGGAAAT
57	gapmer 57	+G*+A*+A*+C*+C*G*T*T*C*A*A*T*C*A*T*A*A*G*+T*+G*+T*+A*+C	ACAGAGGAAGTTGTCTTGAAAACTGGT **GATTTACAACCATTAGAACAAC**CTACTAGTGA
73	gapmer 73	+C*+C*+C*+T*G*G*T*C*A*A*G*G*T*T*A*A*T*+A*+T*+A*+G	TTCATTGTTAATGC**CTATATTAACCTTGACCAGGG**CTTTAA
77	gapmer 77	+G*+T*+A*+G*G*A*G*T*A*A*G*T*T*G*A*T*+C*+T*+G*+C	CCTGTTGCTATTCAT**GCAGATCAACTTACTCCTAC**TTGGC GTGTTTATTCTACAGGTTCTAATGTTTTTCAAACACGT

Bold: actual target of Gapmers.

Columns report the region name (A), the corresponding gapmer name (B), ASO sequence with the chemical modification for the gapmer sequence (C), and the corresponding SARS-CoV-2 target sequence (D), in which the gapmer-matching region is shown in **bold**. The symbol “+” denotes LNA-modified nucleotides, whereas “*” denotes phosphorothioate (PS) linkages.

^a^Locked Nucleic Acid (LNA) modification.

^b^Phosphorothioate (PS) modification.

We screened our gapmers using A549-hACE2 cells, which are lung carcinoma epithelial cells engineered to overexpress human ACE2, and therefore are permissive to SARS-CoV-2 infection.^40^ Cells were transfected with gapmers and then infected with four different SARS-CoV-2 variants, D614G, Delta, Omicron BA.1 (Om BA.1) or Omicron 5 (Om BA.5), at 0.1 multiplicity of infection (MOI). We measured the amount of viral RNA in the cells by performing a qPCR targeting the S protein genomic region. In this assay, 8 out 10 gapmers significantly reduced viral RNA expression (Figure 2A - left panel). We also measured the amount of virus in the supernatant by performing a qPCR targeting the N1 genomic region (Figure 2A - right panel). Gapmers 33, 35, 40, and 41 reduced intracellular and supernatant viral RNA by more than 90% for all four variants (Figure 2A). Similar results were obtained in another cell line, Huh-7.5, with increased permissivity to SARS-CoV-2 infection and replication^41^ (Figure S5B).

**Figure 2:**
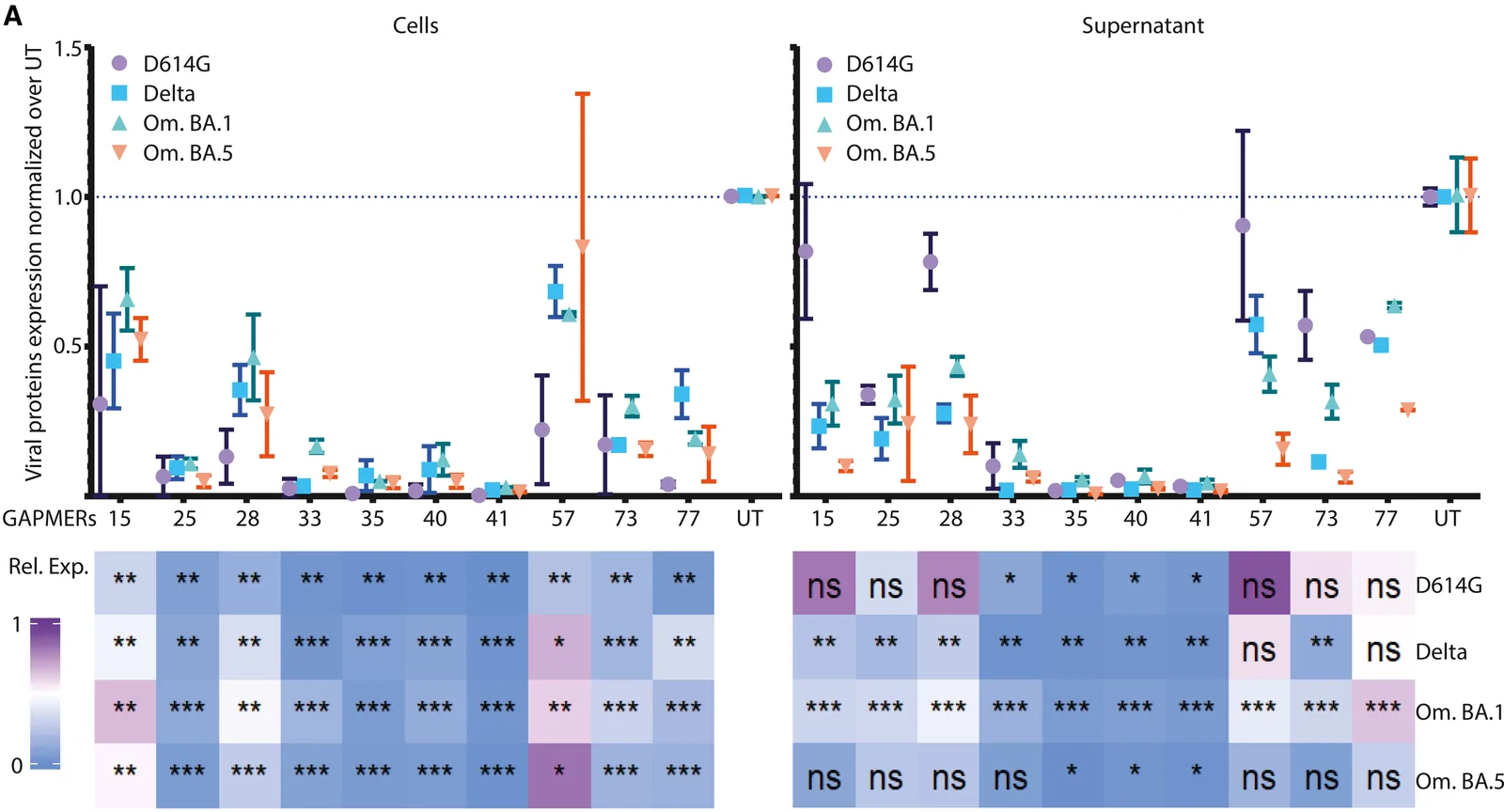
Gapmer targets and efficacy (A) Left panel: qPCR performed on the S genomic region. cDNA was synthesized from intracellular viral RNA isolated from infected A549-hACE2 cells. Right panel: qPCR performed on the Nucleocapsid (N1) genomic region of viral RNA recovered from cell supernatants. Each gapmer was tested in technical triplicates from biological duplicates and normalized to untreated (UT) control. Bars represent mean ± SD of ΔΔCt values relative to UT. Heatmaps below the graphs show relative expression levels of each target compared to UT. Statistical significance is calculated using Linear mixed effects models (LMEs) as indicated in Material and Methods, and is indicated as follows: ns, not significant; ∗, *p* ≤ 0.05; ∗∗, *p* ≤ 10^−3^; ∗∗∗, *p* ≤ 10^−10^. See also Figures S5 and S6.

To visualize the cellular content of viral RNA in cells treated with the gapmers, we performed single molecule FISH (smFISH) with a probe that recognizes the RNA sequence corresponding to the S protein A549-hACE2 cells were treated with gapmers for 12 h (h), infected with the four SARS-CoV-2.^42^ variants and incubated for additional 24 h (Figure 3A). The smFISH images show a reduced level of viral RNA in cells treated with all gapmers (Figures 3A and S6A), corroborating the qPCR results.

**Figure 3:**
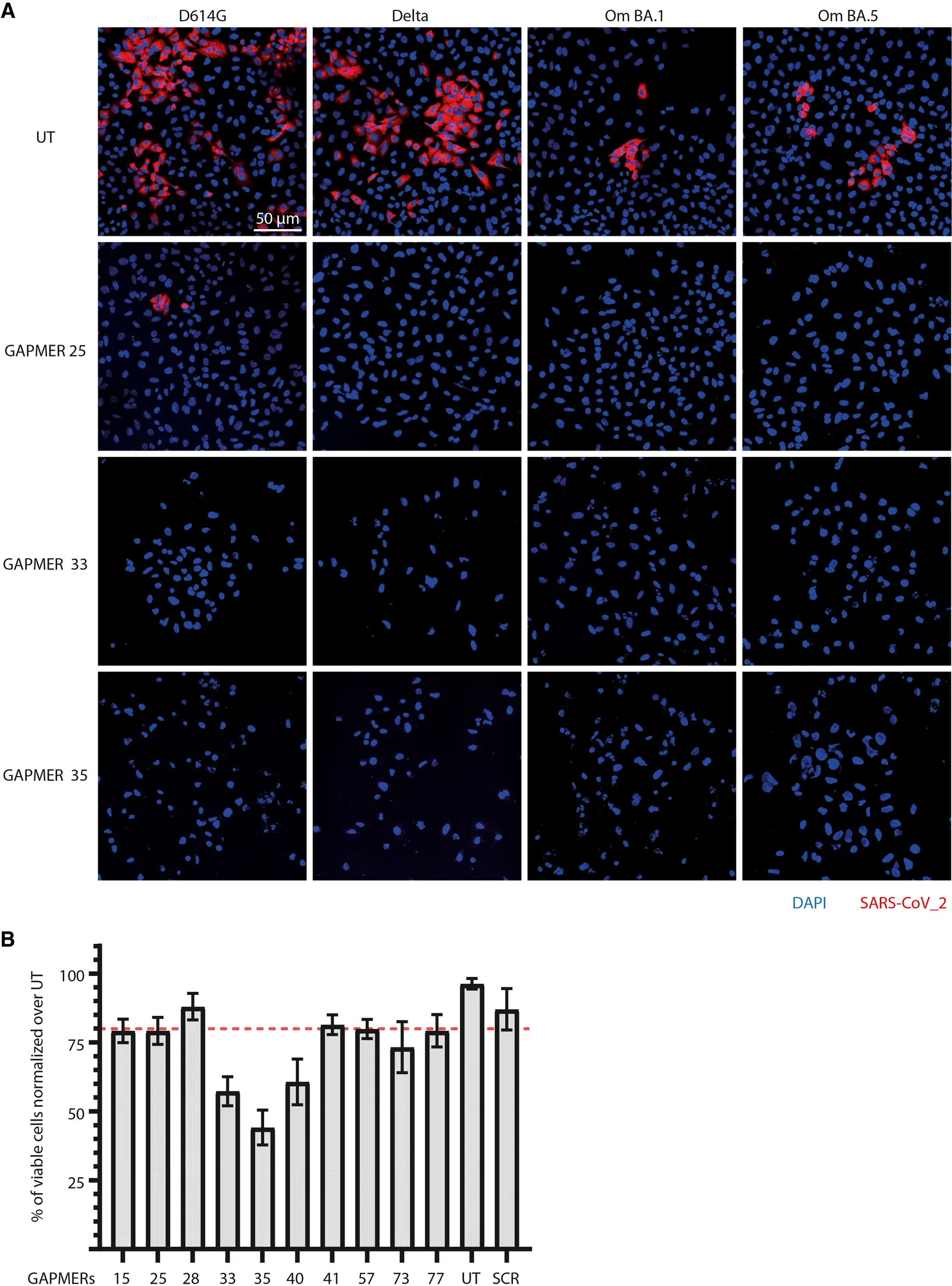
Imaging of viral RNA in treated and untreated cells (A) Representative microscopy images from A549-hACE2 cells. Cells were lipofectamine-transfected with gapmers 25, 33 or 35 and infected with SARS-CoV-2 variants. smFISH was performed using a probe targeting the SARS-CoV-2 S protein (red); nuclei were stained with Hoechst (blue). Scale bar, 50 μm. (B) Viability assay performed by flow cytometry in A549-hACE2 cells transfected with gapmers using lipofectamine. Live cells are double-negative for Annexin V (apoptosis marker) and Propidium Iodide (PI). Graph shows the mean ± SD percentage of viable cells relative to untreated (UT) controls. The cytocompatibility threshold (80%) is indicated by a red dotted line. The experiment was performed in biological triplicates. The y axis represents the percentage of viable cells; the x axis lists the tested gapmers. See also Figure S7.

We next evaluated the cytotoxicity of our gapmers. A549-hACE2 cells were transfected with individual gapmers using Lipofectamine 3000 and cell viability was assessed by flow cytometry using Annexin V and Propidium iodide (PI) staining. Live cells (Annexin V and PI double-negative) were scored using a gating strategy based on positive (H_2_O_2_-treated cells) and negative controls (gapmer scramble, and Lipofectamine-only) (Figure S7A). Flow cytometry demonstrated that Lipofectamine, either alone, with SCR gapmers or with most of our gapmers, induces mild cytotoxicity (Figure S7A). Gapmers 33, 35, and 40 exhibited cytotoxicity in excess of the controls and of the other gapmers, with cell viability below 60% (Figures 3B and S7A), suggesting that these gapmers have either additive or synergic cytotoxicity relative to Lipofectamine alone.

Taken together, our results confirm that 8 of the designed gapmers effectively reduce SARS-CoV-2 RNA levels across multiple viral variants in a cellular system, highlighting their potential as broad-spectrum antiviral agents.

### The viral RNA accessibility to gapmers correlates with the decrease in viral replication

As mentioned above, the design of the pre-ASOs and of the cognate gapmers was based on the SHAPE-MaP analysis of the RNA secondary structure of SARS-CoV-2/Leiden-0002 (GenBank accession: MT510999)^38^; however, no in-silico estimation of the RNA secondary structure of the VoCs was performed. Thus, we performed *in vivo* SHAPE-MaP of the VoCs, and identified the single nucleotide accessibility as reported in Data S2 (Delta), Data S3 (Omicron) and Data S4 (Omicron 5) for the specific region of interest. As previously described^38^ we calculated the average SHAPE reactivity; the results (Table 4) revealed that the structural accessibility of the selected gapmer target regions is highly conserved across SARS-CoV-2 variants. Pairwise comparisons of SHAPE reactivities measured in Delta, Omicron BA.1 and Omicron BA.5 showed strong correlation (R^2^ = 0.844–0.945), indicating that the local structural environments of the target regions are largely maintained (Figure 4A). Furthermore, a statistically significant (*p* ≤ 0.05) correlation was observed between the SHAPE reactivity of each target region and the corresponding gapmer efficacy, in both intracellular and supernatant assays (Figures 3B and 3C). Together with the strong conservation of SHAPE reactivities across variants (Figure 4A), these results indicate that structurally accessible target sites can be identified reproducibly across SARS-CoV-2 variants and represent robust targets for antiviral gapmer design; moreover, gapmer efficacy is maintained across SARS-CoV-2 variants, as expected. These findings experimentally support the hypothesis that RNA structural accessibility is a major determinant of gapmer activity.

**Table 4:** Mean SHAPE reactivities of selected gapmer target regions – related to Figure 4

Sequence name	Mean SHAPE reactivity		
	Delta	Omicron	Omicron5
15	0,3603478	0,39687	0,31613
25	0,7405000	0,66596	0,65550
28	0,4441579	0,37347	0,29611
33	1,2303810	1,06967	1,08790
35	1,1481364	0,92400	0,79918
40	1,1459474	0,98953	0,77689
41	1,0789524	0,88348	0,80529
57	0,5879546	0,56909	0,61923
73	1,1276667	1,00243	0,97943
77	0,4655000	0,60895	0,54030

Columns report the sequence name corresponding to each selected gapmer target region (A), and the mean normalized SHAPE reactivity calculated across the nucleotides of the corresponding target region for the Delta (B), Omicron (C), and Omicron BA.5 (D) variants.

**Figure 4:**
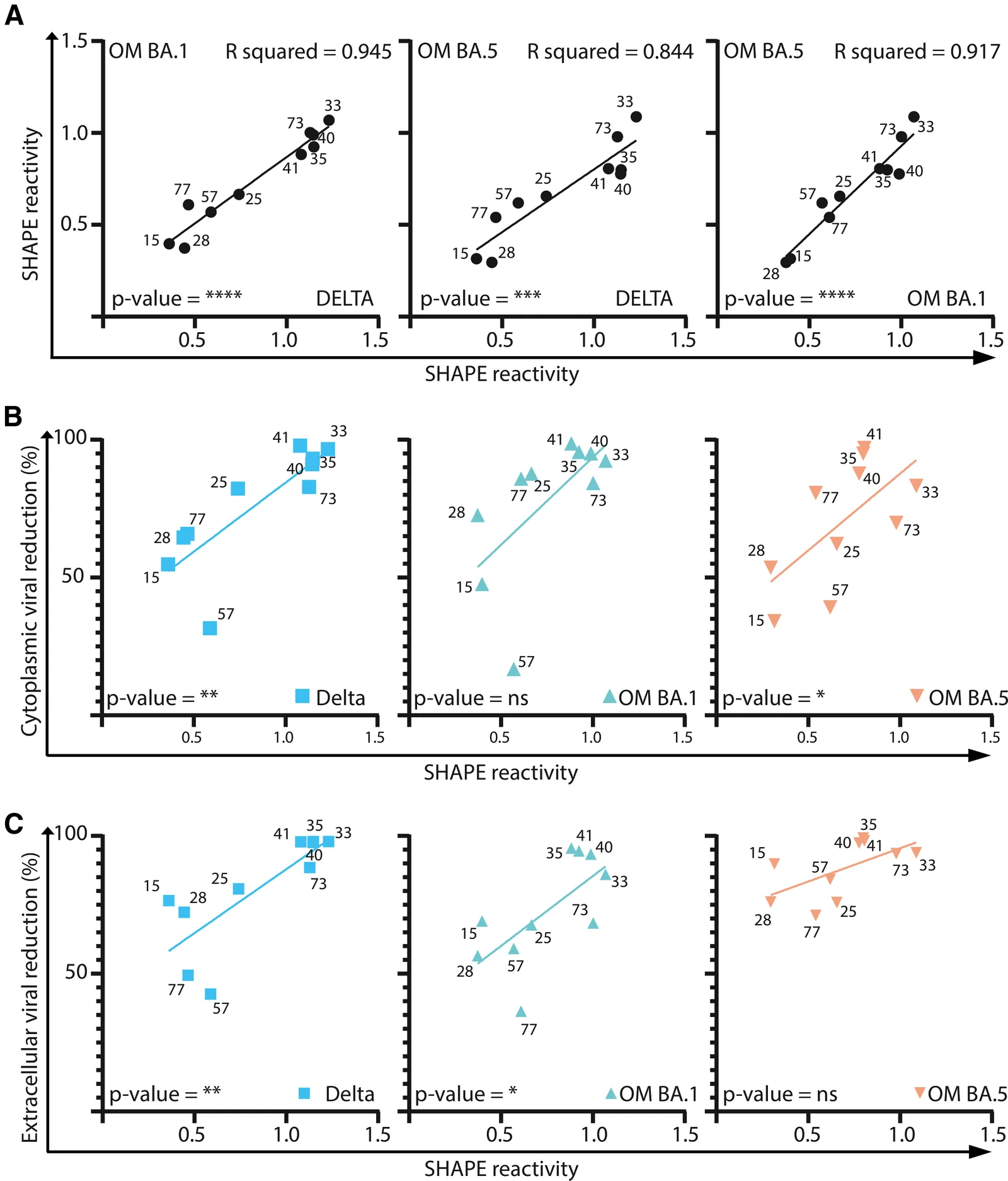
Conservation of target accessibility across SARS-CoV-2 variants and correlation between SHAPE reactivity and gapmer efficacy (A) Pairwise comparison of SHAPE-MaP reactivities of gapmer target regions across SARS-CoV-2 variants of concern (Delta, Omicron BA.1 and Omicron BA.5). Reactivity was measured within infected cells. Each point represents one gapmer target region. Statistical significance is calculated using a two-tailed Pearson’s correlation coefficient and is indicated as follows: ns, not significant; ∗, *p* ≤ 0.05; ∗∗, *p* ≤ 0.01; ∗∗∗, *p* ≤ 0.001; ∗∗∗∗, *p* ≤ 0.0001; b-c) Correlation between SHAPE-MaP reactivity and efficacy of the gapmers; efficacy is measured as the decrease of and intracellular (B) and extracellular (C) RNA produced upon infection. The x axis shows the SHAPE-MaP reactivity values of each target region, whereas the y axis shows the decrease (% inhibition) in intracellular viral RNA measured by QPCR for variants Delta (in blue, left graph), Omicron (in green, middle graph), and Omicron BA.5 (in orange, right graph). Each point corresponds to an individual gapmer target region. Statistical significance is calculated using two-way ANOVA and is indicated as follows: ns, not significant; ∗, *p* ≤ 0.05; ∗∗, *p* ≤ 0.01; ∗∗∗, *p* ≤ 0.001; ∗∗∗∗, *p* ≤ 0.0001.

In conclusion, our pipeline based on RNA secondary structure and sequence conservation has a high rate of success and yields gapmers with robust antiviral activity.

### Gapmers do not activate interferon responses in uninfected cells and decrease interferon responses in infected cells along with the decrease in viral replication

Gapmers can be recognized as nucleic acids released by pathogens and detected by cytosolic DNA recognition pathways, e.g., Toll-like receptors (TLRs), activating an immune response,^43^ including the transcriptional program that results in the production of interferons and proteins encoded by interferon stimulated genes (ISG). Thus, the decrease in viral replication obtained with our gapmers might be due to the activation of the interferon pathway, which indeed affects SARS-CoV-2 replication in cells.^44^

To better understand the host’s response to SARS-CoV-2 variants and to gapmers, we first checked the expression of a panel of Interferon stimulated genes (ISGs) such as ISG54, ISG15, 2′-5′-oligoadenylate synthetase 1 (OAS1) and Interferon Induced Protein with Tetratricopeptide Repeats 1 (IFIT1), in cells infected for 24h with the four selected variants at 0.1 MOI. qPCR performed on cDNA reverse-transcribed from total cellular RNA showed a significant upregulation of the expression of all tested ISGs compared to the uninfected condition, for all viral variants (Figure 5A). This shows that all variants elicit a robust antiviral response.

**Figure 5:**
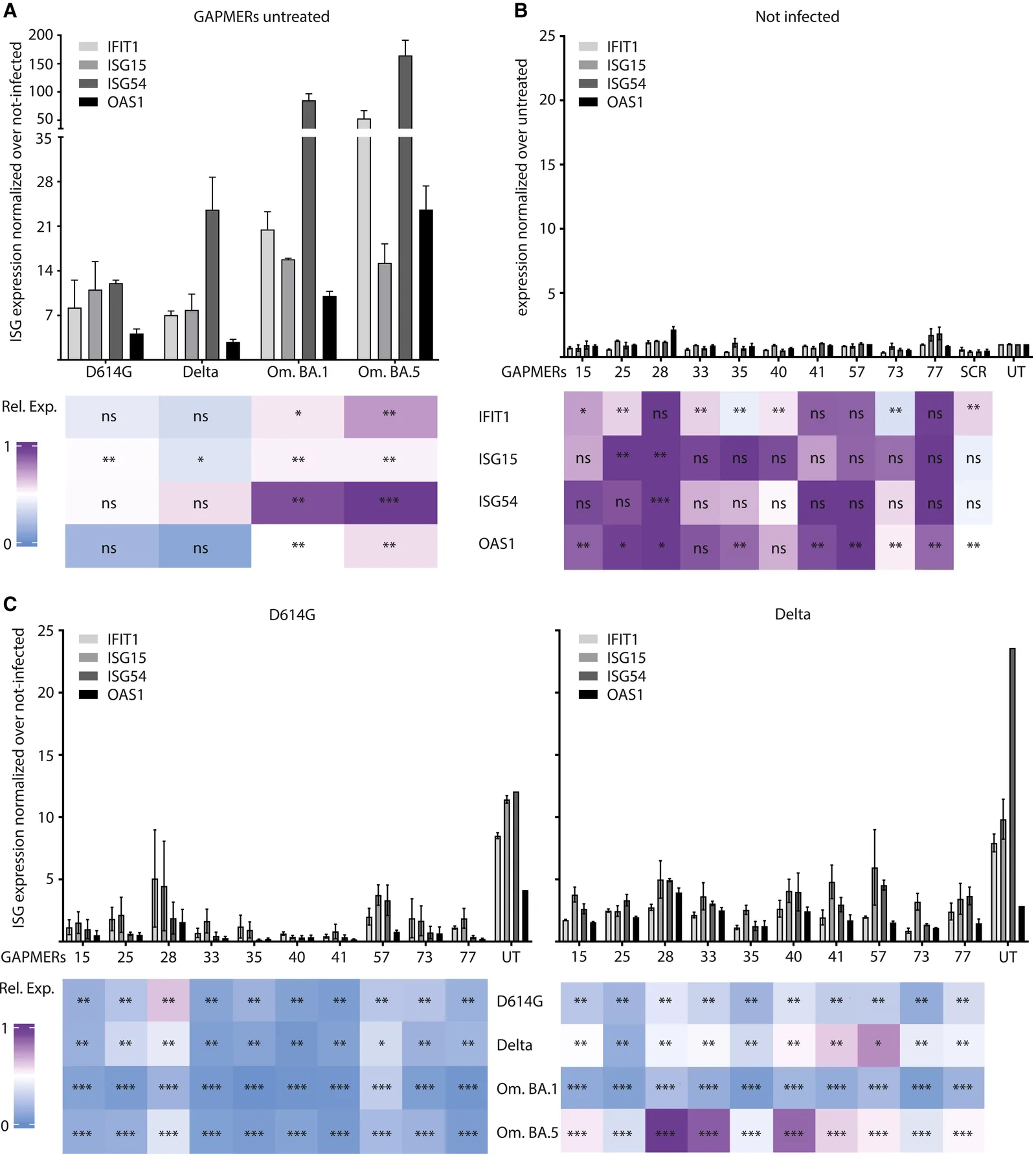
Gapmers do not activate interferon responses in uninfected cells and significantly moderate them in infected cells (A) qPCR of selected ISGs, performed on cDNA synthesized from intracellular viral RNA. A549-hACE2 cells were infected at 0.1 MOI with D614G, Delta, Omicron, or Omicron BA.5 variants. Bar graphs represent the mean ± SD of ΔΔCt values for the expression of ISG54, ISG15, OAS1 and IFIT1 genes, relative to uninfected controls. (B) qPCR of ISGs, performed on cDNA synthesized from intracellular RNA in A549-hACE2 cells transfected with gapmers. Bar graphs represent the mean ± SD of ΔΔCt values for ISG54, ISG15, OAS1, and IFIT1 expression, relative to untreated controls. (C) qPCR of ISGs, performed on cDNA synthesized from intracellular RNA isolated from A549-hACE2 cells infected with variants D614G (left panel) and Delta (right panel). Bar graphs represent the mean ± SD of ΔΔCt values. All experiments were performed in technical triplicates of biological duplicates. Heatmaps below the graphs show relative expression levels of each target compared to control. Statistical significance is calculated using LMEs and post-hoc pairwise comparison, and is indicated as follows: ns, not significant; ∗, *p* ≤ 0.05; ∗∗, *p* ≤ 10^−3;^ ∗∗∗, *p* ≤ 10^−10^. See also Figure S8.

Next, cells were transfected with gapmers using Lipofectamine but were not infected with viruses, and retrotranscribed total cellular RNA 36 h after transfection. Treatment with gapmers elicits a low ISGs response, indicating that our gapmers alone do not activate cytosolic DNA recognition and do not induce antiviral pathways (Figure 5B).

In the final experiment, we combined viral infection and treatment: cells were transfected with gapmers using lipofectamine and infected 12 h later with the four selected SARS-CoV-2 variants at 0.1 MOI. qPCR performed on cDNA from total RNA 24 h post-infection (h p.i.) revealed that treatment with gapmers strongly and significantly reduces the activation of ISGs in infected cells (Figures 5C and S8A), in some cases to levels comparable to non-infected controls.

In brief, our data indicate that gapmers do not activate ISGs, while they decrease the antiviral interferon response of infected cells. Taken together, these data suggest that gapmers cause a decrease in viral replication by directly promoting the destruction of viral RNA, and not indirectly by activating antiviral responses.

### Naked gapmers delivered in mouse lungs are effectively taken up in alveolar macrophages

Since *in vivo* delivery of gapmers with lipofectamine was impractical due to intrinsic toxicity, we rather tested the delivery of naked gapmers to mouse lungs. To track gapmer uptake, Gapmer −35 was labeled with 5′-fluorescein (FAM-35). Anesthetized mice received an intranasal administration of 25 μL of FAM-35 or PBS (negative control) and were sacrificed 24 h later. FAM fluorescence was found in about 2.5% of lung cells in all 3 mice treated with naked FAM-35 (Figure 6A). Thus, our gapmers can be taken up in at least in a small fraction of lung cells.

**Figure 6:**
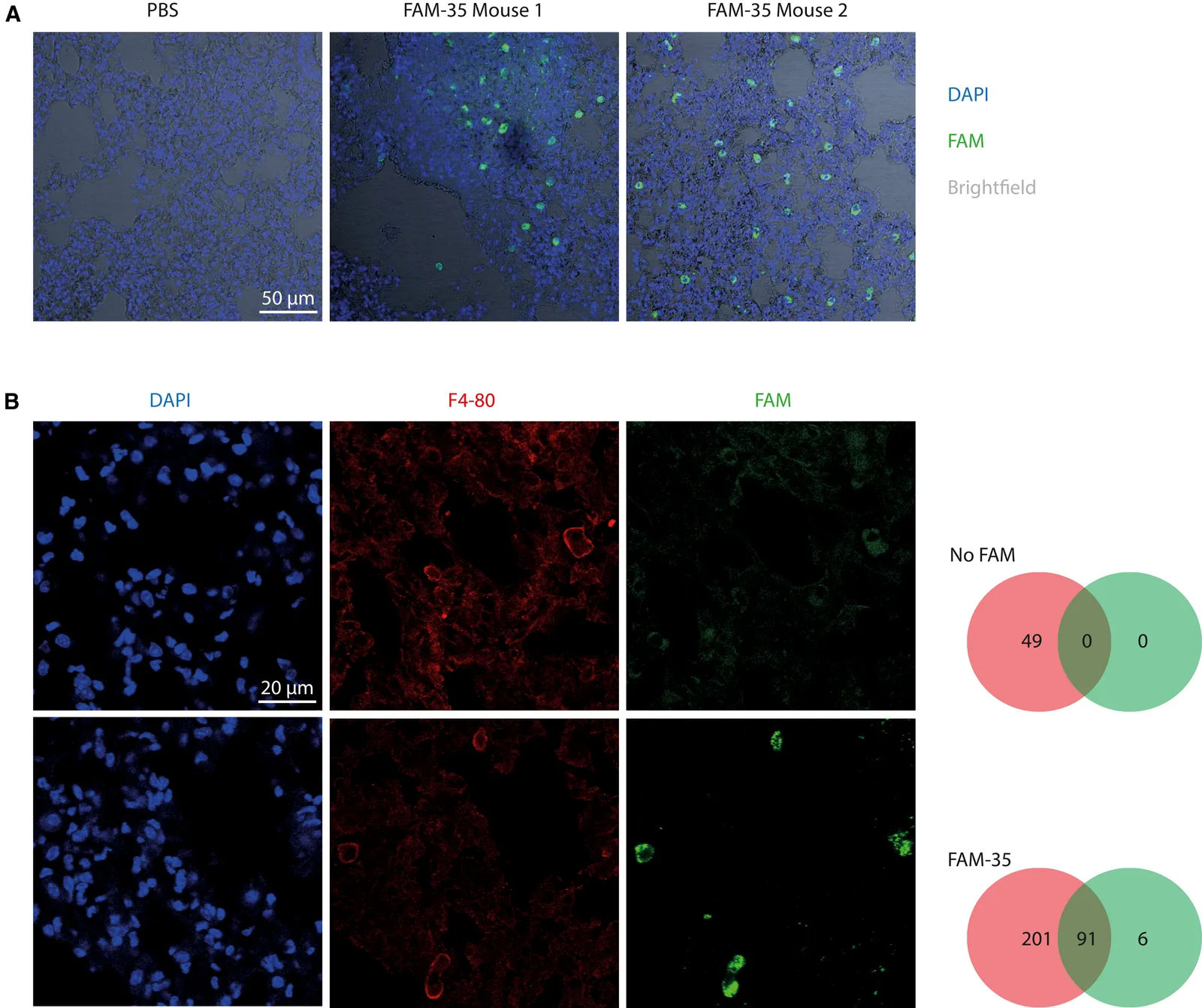
Gapmer delivery in mice lungs (A) Representative microscopy images of lung tissue harvested from PBS-treated or FAM-35-treated mice. Nuclear staining (DAPI, blue) and FAM fluorescence (green) are overlayed on the brightfield image. Scale bar, 50 μm. (B) Representative microscopy images of lung tissue harvested from PBS-treated (upper row) or FAM-35-treated mice (lower row). Left panel: nuclear staining (DAPI, blue), FAM fluorescence (green) and F4/80 macrophage staining (red). Right panel: Venn diagram of PBS-treated (upper row) or FAM-35-treated mice (lower row). Scale bar, 20 μm.

It is well established that alveolar macrophages can uptake SARS-CoV-2 particles, and viral RNA/protein is detected in macrophages in BALF from COVID-19 patients^45^; however, these findings indicate viral uptake rather than productive replication in macrophages.^46^ Accordingly, we aimed to verify that alveolar macrophages are infected by SARS-CoV-2. Lungs sections from the 3 gapmer–treated mice were then stained with a macrophage marker (F4/80) and imaged under the confocal microscope. Across lung sections from both PBS- and gapmer-FAM–treated mice, 341 out of 12,852 total cells (2.6%) were positive for F4/80, a fraction notably similar to the fraction of uninfected cells transfected by naked gapmers (Figure 6B). In lungs from gapmer-FAM–treated mice (lower row), a clear FAM fluorescence signal frequently colocalized with F4/80 staining: 91 out of 97 FAM-positive cells (94%) were F4/80 positive (Venn diagram, Figure 6B). As expected, lungs from PBS-treated mice (upper row) displayed no detectable FAM signal and, consequently, no FAM–macrophage colocalization.

Although gapmer uptake was highly enriched in macrophages, a small fraction of FAM-positive cells lacked F4/80 expression, suggesting that a minor amount of gapmer is internalized by non-macrophage lung cells; however, the identity of these cells was not investigated in the present study.

Collectively, these data demonstrate that naked gapmers are effectively taken up by alveolar macrophages, while their access to other cells is limited. Building on this observation, we took the opportunity to test whether gapmer treatment modulates the macrophage inflammatory programs associated with the cytokine storm characteristic of severe COVID-19.

To define the baseline response of human macrophages, we analyzed cytokine production in human monocyte-derived macrophages (MDMs) differentiated from peripheral blood mononuclear cells (PBMCs) of healthy donors and infected with SARS-CoV-2 at MOI of 10. As shown in Figures 7A and S9A, we assessed cytokine profiles in the supernatant at 24 and 48 h p.i. The results indicated that macrophages responded to viral exposure by releasing a specific set of inflammatory mediators: IL-6 levels were significantly elevated at both 24 and 48 h p.i., while the production of IL-12, IFN-γ, and GM-CSF increased significantly only at 48 h p.i. compared to uninfected controls. In contrast, IL-8, IFN-β, IL-1β, and TNF-α levels remained stable or showed only marginal increases. Notably, viral exposure also induced the secretion of the anti-inflammatory mediator IL-1Ra at 48 h p.i., alongside robust CXCL10 production at both time points. This profile is consistent with an abortive or restrictive infection model, where viral RNA triggers a subset of inflammatory pathways without achieving full productive replication.^16^

**Figure 7:**
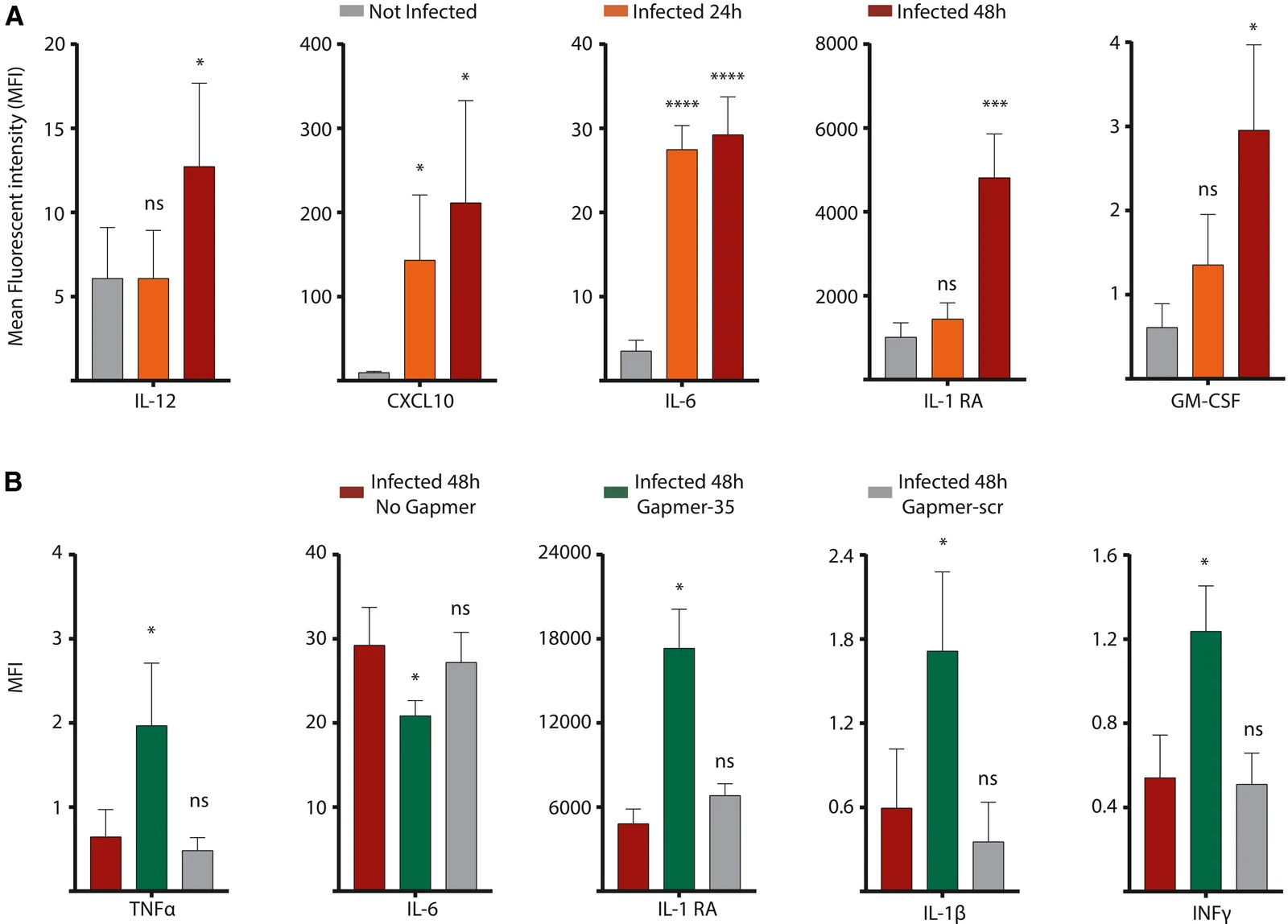
Cytokine profiles in infected human monocyte-derived macrophages (A) Cytokine profiles, measured by Luminex assay, of supernatants from uninfected macrophages (gray), at 24 h. p.i. (orange) and 48 h p.i. (red). (B) Cytokine profiles of supernatants from macrophages at 48 h p.i.; untreated (red), Gapmer-35 treated (green) and scramble treated (gray). a-b) Bar graphs represent the mean ± SD of ΔΔCt values. Statistical significance is calculated using two-sided *t* test versus the relevant control (gray bar) and indicated as follows: ns, not significant; ∗, *p* ≤ 0.05; ∗∗, *p* ≤ 0.01; ∗∗∗, *p* ≤ 10^−3^; ∗∗∗∗, *p* ≤ 10^−4^. See also Figure S9.

We next evaluated whether Gapmer-35 treatment could reprogram this inflammatory signature. As shown in Figures 7B and S9B, control experiments using scramble (SCR)-treated macrophages showed no deviation from the untreated infection profile, confirming that non-specific oligonucleotide exposure does not inherently modulate macrophage activation in this model. In contrast, treatment with gapmer-35 significantly reconfigured the cytokine secretome: while unchallenged infection was characterized by a robust and potentially pathogenic IL-6 response, Gapmer-35 treatment led to a significant decrease in IL-6 levels. Concurrently, we observed a compensatory increase in both IL-1β and its regulatory counterpart IL-1Ra, alongside elevated TNF-α and IL-8 production. The decrease in IL-6, a primary driver of COVID-19-related lung injury, combined with the modulation of cytokines of the IL-1 family, suggests a shift toward a more regulated immune state.

Taken together, these data indicate that a representative gapmer selectively reprograms the inflammatory responses of macrophages infected by SARS-CoV-2, rather than broadly suppress immune responses.

## Discussion

The COVID-19 pandemic and the successive emergence of numerous mutated variant strains of SARS-CoV-2 have highlighted the need for effective therapies targeting multiple viral variants. RNA-based therapeutic strategies have spawned several promising candidate drug classes, including ASOs. Our study meticulously explored the design, evaluation, and optimization of gapmers targeting SARS-CoV-2 RNA to provide a methodological pipeline for potential *in vivo* translation.

The identification of suitable gapmer targets within the SARS-CoV-2 genome required a dual focus on structural accessibility and sequence conservation. RNA secondary structure predictions based on SHAPE-MaP analyses^38^ served as the foundation for selecting single-stranded regions. These regions are essential for viral replication and protein translation^9^ and are therefore likely to be conserved across variants of concern. An initial list of 80 single-stranded regions was narrowed to 24 sequences with a high degree of sequence identity across VoCs including D614G, Delta, Omicron BA.1 and Omicron BA.5. Notably, many of the selected regions encode essential viral proteins, such as the RNA-dependent RNA polymerase (RdRp), N1, and S proteins, but do not match specific regions that have a high mutational rate, for example the receptor binding domain in subunit 1 of the S protein.^47^ This approach yielded sequences that are both single-stranded in the SARS-CoV-2 RNA genome, and therefore accessible to single-stranded complementary oligonucleotides, and highly conserved in different variants, which suggests that they cannot mutate easily. Initial *in vitro* screening revealed that 10 out of 24 pre-ASOs initially selected exhibited >99% efficiency in promoting viral RNA cleavage via RNase H.

We then converted pre-ASOs into gapmers to improve their stability, binding affinity and resistance to nuclease degradation.^48^^,^^49^ Our design included the substitution of phosphorothioates for phosphates in the oligo chain, and the modification to LNA of the 5′ and 3′ ends of the pre-ASOs. Notably, four gapmers (33, 35, 40, 41) demonstrated consistent efficacy across multiple SARS-CoV-2 VoCs in two different cell lines (A549-hACE2 and Huh-7.5). These results highlight the robustness of our design strategy for designing gapmers with broad-spectrum antiviral activity.

The main objective of this study was to experimentally validate the predicted structural accessibility of gapmer target regions in SARS-CoV-2 RNA. Our analysis adopted a targeted SHAPE-MaP approach to interrogate the structural state of the candidate gapmer target regions across multiple SARS-CoV-2 variants. Our data confirmed that these target regions are predominantly single-stranded. A significant correlation between SHAPE reactivity and gapmer efficacy highlights the role of structural accessibility of the viral genome, which can be predicted in silico but also includes RNA-protein interactions and sequence context effects that cannot be easily predicted. Our findings support prior evidence that gapmer performance is shaped by both target properties and cellular environment.^50^

Interestingly, the four gapmers with higher efficacy were all contiguous over the genomic region that encodes the structural proteins (Figure 1C). This collinear configuration has a statistically significant probability of *p* = 7/210 = 0.03 (7 is the number of positions of 4 contiguous segments in a row of 10, and 210 is the number of arrangements containing 4 contiguous segments). Whether this is a statistical fluctuation or has a biological reason is impossible to determine from our data. Thus, the possibility that effective gapmers may consistently target the sequence coding for structural proteins in Coronaviruses should be further evaluated in future studies.

The 6 gapmers with lower antiviral efficacy did not target poorly conserved sequences, but rather regions with less favorable RNA structural organization. In particular, these gapmers targeted regions partially constrained by nearby stem structures, which likely reduce the accessibility of the target sequence to gapmer binding and RNase H recruitment.^51^ Recent studies demonstrated that gapmer:RNA stability depends not only on sequence complementarity, but also on the dynamic accessibility and structural organization of the target RNA region.^52^ Favorable target architecture may combine high local accessibility with structural stability of the surrounding RNA context, whereas completely unstructured or highly dynamic regions may not maintain a sufficiently stable gapmer:RNA duplex for efficient RNase H activity. Importantly, the correlation between SHAPE reactivity and gapmer efficacy across multiple SARS-CoV-2 variants suggests that structural accessibility may represent a conserved functional feature of the viral genome. This indicates that conservation of RNA structure, rather than primary sequence alone, could serve as a robust predictor for broad-spectrum gapmer susceptibility against ssRNA virus.

A possible confounding effect in the effectiveness of gapmer is the activation of cellular antiviral responses, especially because in our experiments gapmers were transfected prior to infection. However, gapmer transfection of uninfected cells showed no activation of ISGs, which is the hallmark of antiviral responses. Indeed, other gapmers like Mipomersen (Kynamro) and Volanesorsen (Waylivra) don’t show significant systemic IFN activation in humans. Infection of cells with SARS-CoV-2 variants significantly upregulated ISGs, indicating the expected robust innate immune activation in infected cells. In cells pretreated with gapmers before infection, ISG expression was much lower than in cells not transfected with gapmers, suggesting that the reduced viral replication and RNA expression leads to a lower host cell response. Mechanistically, this suggests that the antiviral activity of the gapmers depends on the destruction of the viral genome and not on the activation of the host antiviral response. In terms of the therapeutic effects of gapmers against SARS-CoV-2, reducing the activation of IFN pathways might be beneficial, as the patients most affected by SARS-CoV-2 infection show too much IFN and cytokine signaling, leading to a so-called cytokine storm.

The cytokine profile induced by SARS-CoV-2 in human monocyte-derived macrophages (MDMs) reflects a dysregulated inflammatory program consistent with abortive or incomplete pyroptotic signaling.^20^^,^^44^ Although IL-6 and CXCL10 are robustly induced, indicating effective viral RNA sensing, the minimal modulation of IFN-β, IL-1β, and TNF-α suggests only partial engagement of antiviral and inflammasome pathways, even at high MOI. This pattern contrasts with early reports proposing productive macrophage infection and broad cytokine induction, including IFN-β.^53^^,^^54^ Subsequent studies, in fact, demonstrated that primary human MDMs are largely refractory to productive replication supporting a model of restricted or abortive inflammatory activation.^55^^,^^56^

The limited IL-1β maturation, together with delayed IL-1Ra induction, further indicates incomplete inflammasome activation, favoring DAMP release over a canonical IL-1–dominated response. Overall, SARS-CoV-2 appears to initiate early pyroptotic programming in macrophages while attenuating full inflammasome output, a phenotype potentially amplified under high viral exposure.

Gapmer-35 treatment does not broadly suppress but selectively reshapes the inflammatory response of infected macrophages. The marked decrease in IL-6, a key mediator of COVID-19-associated hyperinflammation, indicates targeted attenuation of the pathological NF-κB axis.^57^ Concurrently, the increase in IL-1β and IL-1Ra suggests a more coordinated engagement of inflammasome pathways, shifting macrophages from abortive toward more regulated inflammatory signaling. Importantly, preserved levels of CXCL10, IFN-β, and GM-CSF confirm intact Retinoic acid-inducible gene I - Melanoma differentiation-associated protein 5 (RIG-I/MDA5)-dependent sensing.

Together with our observation that gapmers reduce ISG expression by lowering viral burden rather than stimulating host antiviral pathways, the observation that gapmers rebalance macrophage inflammation suggests that gapmers may represent a promising strategy to mitigate macrophage-driven immunopathology in severe COVID-19.

An important question for future studies concerns the emergence of viral resistance to gapmer treatment. The design strategy described here combines two features that are expected to limit the development of escape variants: structural accessibility and sequence conservation.^39^ By targeting RNA regions that are subject to strong evolutionary constraints, gapmers may induce less resistance-associated mutations than small antiviral molecules. However, direct testing of this hypothesis requires specific *in vitro* approaches and should then be confirmed in clinical trials.

A critical hurdle in therapeutic development of gapmers does remain: their effective delivery into airway and lung cells. Our *in vivo* tests showed that naked gapmers can reach murine lung cells via intranasal delivery, but apart from macrophages the number of transfected cells was low. We confirmed significant cytotoxicity of lipofectamine, which is widely recognized as inadequate for *in vivo* delivery.^58^^,^^59^ We also tested polymers that have been developed for inhalation of small interfering RNA (siRNAs), albeit with unsatisfactory results (not shown). Thus, for therapeutic impact, gapmer delivery to the lung has to be developed.

Overall, this study provides valuable insights into the design and optimization of gapmers as a therapeutic strategy against SARS-CoV-2 and by extension any other ssRNA virus especially for immunodeficient patients. Most notably, our study indicates that the accessibility of conserved single-stranded regions in genomic viral RNA is indeed the primary, albeit not sole, predictor of gapmer efficacy. Targeting those regions is a fast and effective approach that can be tested in cell cultures, with minimal delay between the identification of an emerging virus and the production of gapmers against it. Amid this promising landscape, there is still a prominent bottleneck: the lack of specific delivery systems that are specific or at least effective for inhaled gapmers. Indeed, our results provide a compelling motivation to invest in their development.

### Limitations of the study

This study has several limitations. Gapmer efficacy was primarily assessed in two immortalized cell lines (A549-hACE2 and Huh-7.5) rather than in primary human airway epithelial cultures, which may not fully recapitulate the complexity of the respiratory epithelium *in vivo*. Our screen covered four SARS-CoV-2 variants of concern, and whether the selected target regions remain accessible and conserved in variants that emerge in the future remains to be tested. The *in vivo* experiments in mice demonstrated that naked gapmers can reach lung tissue and are preferentially taken up by alveolar macrophages following intranasal administration, but transfection efficiency in other lung cell types was low, and these studies assessed biodistribution rather than antiviral efficacy or therapeutic outcome in infected animals; an effective, non-toxic pulmonary delivery system for gapmers therefore remains to be developed. The apparent clustering of the most effective gapmers within the genomic region encoding structural proteins reached only borderline statistical significance, and its biological basis, if any, was not established. Finally, the potential for the virus to develop resistance to gapmer treatment through target-site mutation was not experimentally tested and will need to be addressed in future studies.

## Resource availability

### Lead contact

Further information and requests for resources and reagents should be directed to and will be fulfilled by the lead contact, Marco E. Bianchi (bianchi.marco@hsr.it).

### Materials availability

All unique/stable reagents generated in this study are available from the lead contact without restriction.

### Data and code availability

• Data: SHAPE-MaP raw sequencing data have been deposited at the National Center for Biotechnology Information (NCBI) Sequence Read Archive under accession number PRJNA1451745, as listed in the key resources table.
• Code: The original scripts for identification of accessible RNA regions and ASO design is reported in Data S1 and described in the STAR Methods.
• Other items: Any additional information required to reanalyze the data reported in this paper is available from the lead contact upon request.

## Acknowledgments

We thank Sara Morelli for help with statistical analysis in R, Silvia Marchese for experimental support and Francesca Caprioglio for help with the graphical abstract preparation. GG received a PhD Fellowship co-financed with funds from the National Operational Program for Research and Innovation 2014–2020, by FSE REACT-EU funds Action IV.4 “PhD Courses and Research contracts on innovation topics” and Action IV.5 “PhD Courses on Green related topics”.

## Author contributions

Conceptualization, E.M., M.E.B.; methodology, G.G., L.D., L.S.C., V.F., A.L.; Data Analysis, G.G., L.D., L.S.C., A.L., E.M., D.I., D.M.; writing – original draft, G.G., A.DM, M.E.B.; writing – review and editing, G.G., A.DM, M.E.B.; resources, D.I., R.D.F., M.E.B; supervision, A.D.M., E.M., M.E.B.; Funding Acquisition, M.E.B. All authors have read and agreed to this version of the manuscript.

## Declaration of interests

The authors declare no competing interests.

## Declaration of generative AI and AI-assisted technologies in the writing process

During the preparation of this work, the authors used ChatGPT in order to improve language and readability. After using this tool, the authors reviewed and edited the content as needed and take full responsibility for the content of the publication.

## STAR★Methods

### Key resources table

**Table:** 

REAGENT or RESOURCE	SOURCE	IDENTIFIER
**Antibodies**		

CD14 MicroBeads	Miltenyi Biotec	130–050-201 RRID:AB_2665482
F4/80	Thermo Fisher Scientific	MA5-16630 RRID:AB_2538126

**Bacterial and virus strains**		

SARS-CoV-2 variant D614G	GISAID	EPI_ISL_584051
SARS-CoV-2 variant Delta B.1.617.2	GISAID	EPI_ISL_3073880
SARS-CoV-2 variant Omicron BA.1	GISAID	EPI_ISL_10898045
SARS-CoV-2 Omicron subvariant BA.5	GISAID	EPI_ISL_14036188

**Biological samples**		

Human peripheral blood mononuclear cells (PBMCs)	San Raffaele Biobank	BBMRI-ERIC ID: IT_1383758011993577

**Chemicals, peptides, and recombinant proteins**		

APC Annexin-V	BioLegend	640920
Propidium Iodide	Sigma-Aldrich	P4864
TRIzol reagent	Invitrogen	15596026
SuperScript™ IV Reverse Transcriptase	Invitrogen	18090050
SuperScript™ II Reverse Transcriptase	Invitrogen	18064022
SUPERase·In™ RNase Inhibitor (20 U/μL)	Invitrogen	AM2694
RNaseH	Thermo Fisher Scientific	EN0201
Green I Master Mix	Roche Diagnostic	04887352001
TaqPathTM Master Mix	Thermo Fisher Scientific	A15299

**Critical commercial assays**		

Human Magnetic Luminex Assay	R&D Systems	LXSAHM
RNA Clean & Concentrator	Zymo Research	R1015
NucleoMag NGS Clean-up and Size Select Beads	Macherey-Nagel	744970.50
OneTaq® DNA Polymerase	NEB	M0480
NEBNext® Ultra™ II Ligation Module kit	NEB	E7595
NEBNext® Multiplex Oligos for Illum	NEB	E7600
End Prep library kit	NEB	E7546

**Deposited data**		

Shape-Map data raw data	NCBI	PRJNA1451745

**Experimental models: Cell lines**		

A549-hACE2	ATCC	CCL-185
HuH-7.5	JCRB Cell Bank, Osaka	JCRB0403
VERO-E6-TMPRSS2	ATCC	CCL-1587

**Experimental models: Organisms/strains**		

hyACE2 C57BL/6 female mice	Generated in San Raffaele Scientific Institute	Fumagalli et al.^60^

**Oligonucleotides**		

ASO_1_For ACCAACCAACTTTCGATCTCT	EUROFINS	This Work
ASO_5_For AAGACGGCAGTGAGGACAAT	EUROFINS	This Work
ASO_12_For CGTCTGCGGTATGTGGAAAG	EUROFINS	This Work
ASO_14_For CTGACGATGCTGTTGTGTGT	EUROFINS	This Work
ASO_15_For ATCAAGAATCCTAGGGGCCG	EUROFINS	This Work
ASO_16_For GCAATGCTCCAGGTTGTGAT	EUROFINS	This Work
ASO_17_For CCACCACTTAACCGAAATTATGT	EUROFINS	This Work
ASO_20_ForTCCAGACATGTTCCTCGGAA	EUROFINS	This Work
ASO_25_For TCCAGACATGTTCCTCGGAA	EUROFINS	This Work
ASO_28_For GCCTTGAAGCCCCTTTTCTC	EUROFINS	This Work
ASO_29_For TACTGCGCTTCGATTGTGTG	EUROFINS	This Work
ASO_33_For TCTTTCAGACTGTTTGCGCG	EUROFINS	This Work
ASO_35_For GTTGCTACATCACGAACGCT	EUROFINS	This Work
ASO_40_For ATGGTGCTAACAAAGACGGC	EUROFINS	This Work
ASO_41_For TGCTTGACAGATTGAACCAGC	EUROFINS	This Work
ASO_44_For CAGACCACACAAGGCAGATG	EUROFINS	This Work
ASO_57_For CGAGCAACATAAGCCCGTTA	EUROFINS	This Work
ASO_61_For CCATCTCACTTGCTGGTTCC	EUROFINS	This Work
ASO_66_For AGTCCTCAGAATACAAAGGTCCT	EUROFINS	This Work
ASO_68_For GGTGTTTAAACCGTGTTTGTACT	EUROFINS	This Work
ASO_69_For TGCCGACTACTATAGCAAAGAA	EUROFINS	This Work
ASO_73_For TTCATACAAACCACGCCAGG	EUROFINS	This Work
ASO_77_For TCTTTTGGTGGTGTCAGTGT	EUROFINS	This Work
ASO_81_For GCCTTGAAGCCCCTTTTCTC	EUROFINS	This Work
Spike_For GTCCTTCCCTCAGTCAGCAC	EUROFINS	This Work
GAPDH_For TCGTGGAAGGACTCATGACC	EUROFINS	This Work
hISG54-For ATGTGCAACCTACTGGCCTAT	EUROFINS	This Work
hISG15-For TCCTGGTGAGGAATAACAAGGG	EUROFINS	This Work
hOAS1-For GATCTCAGAAATACCCCAGCCA	EUROFINS	This Work
hIFIT1-For AGTGTGGGAATACACAACCTACT	EUROFINS	This Work
Library For #6 TCTCAACCTGTGCTTGTGAAATTG	EUROFINS	This Work
Library For #34 TCAAGATCTCAATGGTAACTGGTATGA	EUROFINS	This Work
Library For #38 GGTCTAGTGGCTAGCATAAAGAACT	EUROFINS	This Work
Library For #56 CAGGGTGTTAACTGCACAGAAGTC	EUROFINS	This Work
Library For #61 TTATTGTTGGCGTTGCACTTCTT	EUROFINS	This Work
Library For #64 AATGGCTTGTCTTGTAGGCTTGAT	EUROFINS	This Work
Library For #68 AAGGCGTTCCAATTAACACCAATAG	EUROFINS	This Work
Library For #69 CGGTGATGCTGCTCTTGCTT	EUROFINS	This Work
ASO_1_Rev CTGATGATCGGCTGCAACAC	EUROFINS	This Work
ASO_5_Rev GCTTCTTCCACAATGTCTGCA	EUROFINS	This Work
ASO_12_Rev TAAGACGGGCTGCACTTACA	EUROFINS	This Work
ASO_14_Rev AGGTCAGTCTCAGTCCAACA	EUROFINS	This Work
ASO_15_Rev TGAAAGACATCAGCATACTCCTG	EUROFINS	This Work
ASO_16_Rev TGTCCAGTCACATGTTGCAA	EUROFINS	This Work
ASO_17_Rev CAGCATCACCATAGTCACCTTTT	EUROFINS	This Work
ASO_20_Rev TTTGTGGCCTGTTAATTGCAG	EUROFINS	This Work
ASO_25_Rev TGACATAGTGTAGGCAATGATGG	EUROFINS	This Work
ASO_28_Rev TCGTAACAATTAGTATGCCAGCA	EUROFINS	This Work
ASO_29_Rev TAGTACCGTTGGAATCTGCC	EUROFINS	This Work
ASO_33_Rev CAGGTCCTTGATGTCACAGC	EUROFINS	This Work
ASO_35_Rev ACCTGAAAGTCAACGAGATGAA	EUROFINS	This Work
ASO_40_Rev AGGAACGAGAAGAGGCTTGA	EUROFINS	This Work
ASO_41_Rev AATTTCCTTGGGTTTGTTCTGG	EUROFINS	This Work
ASO_44_Rev GGCTCTTTCAAGTCCTCCCT	EUROFINS	This Work
ASO_57_Rev TCATGCCTCTAAAAGCCCCA	EUROFINS	This Work
ASO_61_Rev TCTAGGTGGAATGTGGTAGGA	EUROFINS	This Work
ASO_66_Rev TGGATATGGTTGGTTTGGTACA	EUROFINS	This Work
ASO_68_Rev AGCCTCTAGACAAAATTTACCGA	EUROFINS	This Work
ASO_69_Rev AACACCTAAAGCAGCGGTTG	EUROFINS	This Work
ASO_73_Rev GAGTTTTAACCTCTCTTCCGTGA	EUROFINS	This Work
ASO_77_Rev GCCCCTATTAAACAGCCTGC	EUROFINS	This Work
ASO_81_Rev GCATTTCCAGCAAAGCCAAA	EUROFINS	This Work
Spike_Rev ATGGCAGGAGCAGTTGTGAA	EUROFINS	This Work
GAPDH_Rev TGAGAGTCGGCCCATGTGATA	EUROFINS	This Work
hISG54-Rev TGAGAGTCGGCCCATGTGATA	EUROFINS	This Work
hISG15-Rev GTCAGCCAGAACAGGTCGTC	EUROFINS	This Work
hOAS1-Rev AGCTACCTCGGAAGCACCTT	EUROFINS	This Work
hIFIT1-REV GGTCACCAGACTCCTCACATTT	EUROFINS	This Work
Library Rev #6 GCCTTTGAGTGTGAAGGTATTGTTT	EUROFINS	This Work
Library Rev #34 CAGCAGCATACACAAGTAATTCCTT	EUROFINS	This Work
Library Rev #38 ATGAAGTCTGTGAATTGCAAAGAACA	EUROFINS	This Work
Library Rev #56 TTGTCTTGTTCAACAGCTATTCCAG	EUROFINS	This Work
Library Rev #61 ACAGCTGGTAATAGTCTGAAGTGAA	EUROFINS	This Work
Library Rev #64 TTAGTAATATCTCTGCTATAGTAACCTGAA	EUROFINS	This Work
Library Rev #68 CTCAAGCTGGTTCAATCTGTCAAG	EUROFINS	This Work
Library Rev #69 TGTCTCTGCGGTAAGGCTTGA	EUROFINS	This Work

**Software and algorithms**		

Fiji	Schindelin et al.^61^	RRID:SCR_002285; fiji.sc
ImageJ	National Institutes of Health	RRID:SCR_003070; imagej.net
Cellpose	Stringer et al.^62^	RRID:SCR_021716; cellpose.org
RStudio	Posit Software, PBC	RRID:SCR_000432; posit.co
FlowJo	BD Biosciences	RRID:SCR_008520; flowjo.com
BioRender	BioRender.com	RRID:SCR_018361; biorender.com

### Experimental model and study participant details

#### Ethics statement

Human PBMCs were obtained from the Biological Resource Center (Centro Risorse Biologiche, CRB) of the IRCCS Ospedale San Raffaele (Milan, Italy). PBMC were isolated from blood samples obtained from 3 healthy donors provided written informed consent for the collection and use of biological specimens for research purposes, in accordance with the Declaration of Helsinki, The biobanking protocol was approved by the Institutional Review Board/Ethics Committee of the IRCCS Ospedale San Raffaele under the protocol BBMRI-ERIC ID: IT_1383758011993577.

A fully anonymized form to protect donor privacy, in compliance with institutional review board guidelines and GDPR regulations. Consequently, demographic characteristics such as age, sex/gender, and ancestry were unavailable to the investigators, and sex/gender-based stratification could not be performed.

Experiments with mice were performed with hyACE2 knock-in mice.^60^ Six hyACE2 C57BL/6 female at 8–10 weeks of age were used. All experimental procedures were approved by Italian Ministry of Health (authorization 270/2022-PR).

All other experiments were performed using established cell lines (A549-hACE2, HuH-7.5, VERO-E6-TMPRSS2) obtained from commercial or academic sources. Cell lines obtained from commercial suppliers were authenticated and routinely tested for mycoplasma contamination by the providers. Cell lines received from collaborating laboratories were authenticated and routinely tested for mycoplasma contamination by the respective laboratories. In addition, all cell lines were weekly tested for mycoplasma contamination in our laboratory.

#### Cell culture

The A549-hACE2 and HuH-7.5 cell lines were purchased from NCBI (ATCC number: CCL-185) and Japanese Collection of Research Bioresources (JCRB) Cell Bank, Osaka, Japan (Ref: JCRB0403), respectively. The former were cultured in RPMI 1640 Medium (Gibco, ref. 21875-034), supplemented with 10% fetal bovine serum (FBS, Gibco), 1% Penicillin-Streptomycin (Pen/Strep; Gibco, ref. 15140-122), 1% L-Glutamine 200 mM (Gibco, ref. 25030-024), 1% Non-Essential Amino Acids (MEM NEAA; Gibco, ref. 11140-050), 1% Sodium Pyruvate (Gibco, ref. 11360-070). The latter in Dulbecco’s modified Eagle’s medium (DMEM + GlutaMAX ; Gibco, ref. 61965-026) supplemented with 10% fetal bovine serum 1% Pen/Strep, 1% of MEM NEAA, and 1% Sodium Pyruvate. All cells were cultured at 37°C and 95–99% humidity, in an atmosphere containing 5% CO2.

#### Mice

Mice had *ad libitum* access to drinking water and chow (VRF1 standard diet, Safe, 801900). Mice were housed under specific pathogen-free conditions with a 12-h light/12-h dark cycle, a temperature ranging between 20 °C and 23 °C and 60% humidity.

### Method details

#### Cell treatment

HuH-7.5 or A549-hACE2 cells were plated in complete medium. After 24h cells were treated with 100 nM Gapmers and LIPOFECTAMINE 3000 according to manufacturer’s protocol. Infection: 12h after treatment the medium was replaced with DMEM supplemented with 2% FBS and the cells were infected with 0.1 MOI SARS-CoV-2 variants D614G, Delta, Omicron and Omicron 5 according. MOI was calculated as follows: (MOI x no. cell plated)/(viral concentration PFU/ML) x 1000.

#### Virus preparation

VERO-E6-TMPRSS2 cells were purchased from NCBI (ATCC number: CRL-1587). Cells were cultured in DMEM, supplemented with 2% FBS,1% Pen/Strep, 1% MEM NEAA, 1% Sodium Pyruvate. After 24 h, cells were inoculated with 0.001 or 0.01 MOI of SARS-CoV-2 variant D614G (GISAID ID: EPI_ISL_584051), SARS-CoV-2 variant Delta B.1.617.2 (GISAID ID: EPI_ISL_3073880), SARS-CoV-2 variant Omicron BA.1 (GISAID ID: EPI_ISL_10898045) and SARS-CoV-2 Omicron subvariant BA.5 (GISAID ID: EPI_ISL_14036188). The supernatant was collected after 48–72h; 200 μL were kept for titration and the rest centrifuged at 5000 g for 5 min. The pellet was resuspended in an appropriate volume, aliquoted and stored at −80°C. Virus stocks were titrated by plaque assay using Vero E6-TMPRSS as described in.^63^

#### Viral RNA extraction

SARS-CoV-2 RNA (D614G variant) was extracted from the supernatant of VERO-E6 cells infected at MOI 0.1. The supernatant was harvested 48 h p.i. and centrifuged to remove debris. The supernatant was mixed with 5X PEG-it virus precipitation solution (System Bioscience, Cat. N. LV810A-1), vortexed and incubated overnight at 4°C. The supernatant/PEG-it mixture was centrifuged, transferred to a fresh tube and centrifuged again. The samples were lysed with TRIzol reagent (Invitrogen, Cat. N. 15596026) for RNA extraction, resuspend in RNAse-free water and stored at −20°C. RNA was quantified using a NanoDrop spectrophotometer, obtaining a concentration of 25 ng/μL. Samples were stored at −80°C prior to use. All experiments that involved active virus handling were carried out in the Bio Safety Level 3 (BLS-3) room in INGM.

#### *In vitro* RNA folding

500 ng of viral RNA were denatured at 95°C for 2 min, followed by rapid cooling on ice for 1 min, followed by incubation with folding buffer containing 50 μM HEPES pH 7.5, 100 mM KCl at 37°C for 15 min 10 mM MgCl2 was added, and the sample was incubated for an additional 15 min at 37°C. Subsequently the folded RNA was hybridised with ASOs at varying final concentrations (0.1 μM, 1 μM, 5 μM, 10 μM) by incubating at 37°C for 15 min. The ASO-RNA solution was incubated with 2.5U of RNaseH (Thermo Fisher, Cat. N. EN0201) diluted in 20 mM Tris-HCl pH 7.5, 100 mM KCl, 10 mM MgCl2, 0.1 mM DTT, 4.7% sucrose. The digested RNA was reverse transcribed into cDNA using the SSIV Reverse Transcriptase (Invitrogen, ref. 18090050) according to the manufacturer’s protocol.

#### Total RNA extraction

24 h p.i. the supernatants from each condition were mixed ViRNAExTM solution (Metacell s.r.o. Ref. 111560) according to the protocol and cells were lysed with TRIzol reagent for RNA extraction. The samples were stored at −20°C until RNA extraction, performed following the manufacturer’s protocol. After collection of the aqueous phase, an equal volume of 98% ethanol was added and the RNA cleanup was per-formed using the RNA Clean & Concentrator kit (Zymo Research; Ref: R1015) following the manufacturer’s protocol. RNA was eluted in 12 μL of DNase/RNAse-free water and quantified using a NanoDrop spectrophotometer.

#### qPCR

cDNA was diluted 1:150 in RNase-free water. qPCR was performed on RTcDNA using the primers reported in Table 5. The reaction mix was prepared according to LightCycler 480 SYBR Green I master (Roche Diagnostic, ref. 04887352001) or TaqPathTM Master Mix (Thermo Fisher Scientific; Ref: A15299) following the manufacturers’ protocols. The reactions were carried out using the Light-Cycler480 Instrument II (Roche) or QuantStudio 5 real-time PCR systems, respectively. Cycle thresholds (Cts) were normalised over the housekeeping gene (GAPDH) and ΔΔCt values were calculated relative to untreated controls. Cts obtained from extracellular RNA gapmers treatment were normalised over the non-treated sample.

**Table 5:** Primer list – related to STAR Methods

Primer list				
Forward primer		Reverse primer		Target
Name	Sequence	Name	Sequence	
ASO_1_For	ACCAACCAACTTTCGATCTCT	ASO_1_Rev	CTGATGATCGGCTGCAACAC	ASO 1 target genomic region (5′ UTR)
ASO_5_For	AAGACGGCAGTGAGGACAAT	ASO_5_Rev	GCTTCTTCCACAATGTCTGCA	ASO 5 target genomic region (POL)
ASO_12_For	CGTCTGCGGTATGTGGAAAG	ASO_12_Rev	TAAGACGGGCTGCACTTACA	ASO 12 target genomic region (POL)
ASO_14_For	CTGACGATGCTGTTGTGTGT	ASO_14_Rev	AGGTCAGTCTCAGTCCAACA	ASO 14 target genomic region (POL)
ASO_15_For	ATCAAGAATCCTAGGGGCCG	ASO_15_Rev	TGAAAGACATCAGCATACTCCTG	ASO 15 target genomic region (POL)
ASO_16_For	GCAATGCTCCAGGTTGTGAT	ASO_16_Rev	TGTCCAGTCACATGTTGCAA	ASO 16 target genomic region (HEL)
ASO_17_For	CCACCACTTAACCGAAATTATGT	ASO_17_Rev	CAGCATCACCATAGTCACCTTTT	ASO 17 target genomic region (HEL)
ASO_20_For	TCCAGACATGTTCCTCGGAA	ASO_20_Rev	TTTGTGGCCTGTTAATTGCAG	ASO 20 target genomic region (HEL)
ASO_25_For	AGGCTGTTTAATAGGGGCTGA	ASO_25_Rev	TGACATAGTGTAGGCAATGATGG	ASO 25 target genomic region (S1)
ASO_28_For	GCCTTGAAGCCCCTTTTCTC	ASO_28_Rev	TCGTAACAATTAGTATGCCAGCA	ASO 28 target genomic region (ORF3A)
ASO_29_For	TACTGCGCTTCGATTGTGTG	ASO_29_Rev	TAGTACCGTTGGAATCTGCC	ASO 29 target genomic region (ENV)
ASO_33_For	TCTTTCAGACTGTTTGCGCG	ASO_33_Rev	CAGGTCCTTGATGTCACAGC	ASO 33 target genomic region (MEMB)
ASO_35_For	GTTGCTACATCACGAACGCT	ASO_35_Rev	ACCTGAAAGTCAACGAGATGAA	ASO 35 target genomic region (MEMB)
ASO_40_For	ATGGTGCTAACAAAGACGGC	ASO_40_Rev	AGGAACGAGAAGAGGCTTGA	ASO 40 target genomic region (NP)
ASO_41_For	TGCTTGACAGATTGAACCAGC	ASO_41_Rev	AATTTCCTTGGGTTTGTTCTGG	ASO 41 target genomic region (NP)
ASO_44_For	CAGACCACACAAGGCAGATG	ASO_44_Rev	GGCTCTTTCAAGTCCTCCCT	ASO 44 target genomic region (ORF10)
ASO_57_For	CGAGCAACATAAGCCCGTTA	ASO_57_Rev	TCATGCCTCTAAAAGCCCCA	ASO 57 target genomic region (NSP2)
ASO_61_For	CCATCTCACTTGCTGGTTCC	ASO_61_Rev	TCTAGGTGGAATGTGGTAGGA	ASO 61 target genomic region (PL-PRO)
ASO_66_For	AGTCCTCAGAATACAAAGGTCCT	ASO_66_Rev	TGGATATGGTTGGTTTGGTACA	ASO 66 target genomic region (PL-PRO)
ASO_68_For	GGTGTTTAAACCGTGTTTGTACT	ASO_68_Rev	AGCCTCTAGACAAAATTTACCGA	ASO 68 target genomic region (PL-PRO)
ASO_69_For	TGCCGACTACTATAGCAAAGAA	ASO_69_Rev	AACACCTAAAGCAGCGGTTG	ASO 69 target genomic region (PL-PRO)
ASO_73_For	TTCATACAAACCACGCCAGG	ASO_73_Rev	GAGTTTTAACCTCTCTTCCGTGA	ASO 73 target genomic region (POL)
ASO_77_For	TCTTTTGGTGGTGTCAGTGT	ASO_77_Rev	GCCCCTATTAAACAGCCTGC	ASO 77 target genomic region (S1)
ASO_81_For	GCCTTGAAGCCCCTTTTCTC	ASO_81_Rev	GCATTTCCAGCAAAGCCAAA	ASO 81 target genomic region (ORF3A)
Spike_For	GTCCTTCCCTCAGTCAGCAC	Spike_Rev	ATGGCAGGAGCAGTTGTGAA	Spike genomic region
GAPDH_For	TCGTGGAAGGACTCATGACC	GAPDH_Rev	ATGATGTTCTGGAGAGCCCC	GAPDH genomic region
hISG54_For	ATGTGCAACCTACTGGCCTAT	hISG54_Rev	TGAGAGTCGGCCCATGTGATA	Human ISG54 genomic region
hISG15_For	TCCTGGTGAGGAATAACAAGGG	hISG15_Rev	GTCAGCCAGAACAGGTCGTC	Human ISG15 genomic region
hOAS1_For	GATCTCAGAAATACCCCAGCCA	hOAS1_Rev	AGCTACCTCGGAAGCACCTT	Human OAS1 genomic region
hIFIT1_For	AGTGTGGGAATACACAACCTACT	hIFIT1_Rev	GGTCACCAGACTCCTCACATTT	Human IFIT1 genomic region
Library For #6	TCTCAACCTGTGCTTGTGAAATTG	Library Rev #6	GCCTTTGAGTGTGAAGGTATTGTTT	ASO 57 target genomic region
Library For #34	TCAAGATCTCAATGGTAACTGGTATGA	Library Rev #34	CAGCAGCATACACAAGTAATTCCTT	ASO 73 target genomic region
Library For #38	GGTCTAGTGGCTAGCATAAAGAACT	Library Rev #38	ATGAAGTCTGTGAATTGCAAAGAACA	ASO 15 target genomic region
Library For #56	CAGGGTGTTAACTGCACAGAAGTC	Library Rev #56	TTGTCTTGTTCAACAGCTATTCCAG	ASO 25 and 77 target genomic region
Library For #61	TTATTGTTGGCGTTGCACTTCTT	Library Rev #61	ACAGCTGGTAATAGTCTGAAGTGAA	ASO 28 target genomic region
Library For #64	AATGGCTTGTCTTGTAGGCTTGAT	Library Rev #64	TTAGTAATATCTCTGCTATAGTAACCTGAA	ASO 33 and 35 target genomic region
Library For #68	AAGGCGTTCCAATTAACACCAATAG	Library Rev #68	CTCAAGCTGGTTCAATCTGTCAAG	ASO 40 target genomic region
Library For #69	CGGTGATGCTGCTCTTGCTT	Library Rev #69	TGTCTCTGCGGTAAGGCTTGA	ASO 41 target genomic region

Columns report the forward primer name (A) and sequence (5′–3′) (B), the reverse primer name (C) and sequence (5′–3′) (D), and the corresponding target gene or genomic region (E).

For, forward; Rev, Reverse; GAPDH, Glyceraldehyde-3-Phosphate Dehydrogenase; hISG, human Interferon-Stimulated Gene; hOAS, human 2′-5′-Oligoadenylate Synthetase; hIFIT, human Interferon-Induced Protein; UTR, Untranslated Region; POL, Polymerase; HEL, Helicase; S1, Spike Subunit 1; ORF, Open Reading Frame; ENV, Envelope; MEMB, Membrane; NP, Nucleoprotein; NSP, Non-Structural Protein; PL-PRO, Papain-Like Protease.

#### Macrophages isolation, infection and analysis

Buffy coats were obtained from anonymous healthy donors. Human peripheral blood mononuclear cells (PBMCs) were isolated via Ficoll-Paque density gradient centrifugation, and CD14^+^ monocytes were subsequently purified through positive immunomagnetic selection using CD14 MicroBeads (Miltenyi Biotec, ref. 130–050-201) according to the manufacturer’s instructions. The isolated CD14^+^ monocytes were seeded in 24-well plates and cultured in RPMI 1640 medium (Gibco) supplemented with 10% fetal bovine serum (FBS; Gibco), 1% penicillin-streptomycin, 1% L-glutamine (200 mM), 1% non-essential amino acids, and 1% sodium pyruvate. Macrophage differentiation was induced by the addition of human Macrophage Colony Stimulating Factor (M-CSF; PeproTech, ref. 300-25) at a concentration of 20 ng/mL. After 6 days of differentiation, the M-CSF stimulus was removed, and the resulting macrophages were treated with 2.5 μM of Gapmer-35, a scrambled Gapmer control (SCR), or PBS. Following a 12-h incubation, the macrophages were infected with the SARS-CoV-2 Delta variant at a MOI of 10. Cell culture supernatants were harvested at 24 and 48 h p.i. Cytokine and chemokine profiles in the supernatants were characterized using a custom Human Magnetic Luminex Assay (R&D Systems, Ref: LXSAHM) according to the manufacturer’s protocol and analyzed on a Luminex 200 system.

#### Imaging

A549 cells (5x104) were plated onto 15 mm glass coverslips in a 24-well plate and treated/infected as reported above. After 36h medium was removed, and cells were washed twice with PBS. Subsequently, cells were fixed with 4% PFA for 15 min at room temperature (RT).

For the mice lung section: 100 μM FAM-35 or PBS were intranasally administered into mice, under gaseous anesthesia (2–5% isoflurane). After 24h, the mice were sacrificed and lungs were collected. The lungs were left in 4% PFA overnight, washed with PBS, and incubated in 30% sucrose for at least 8h. After incubation, the lungs were embedded in OCT and stored at −80°C. The lungs were sliced into 20 μm sections and placed on glass slides. Lung slides were blocked 1 h at RT in 10% Goat Serum/PBS 1X and stained ON at 4°C with F4/80 primary antibody (Thermo Fisher Scientific, Ref: MA5-16630) diluted 1:100 in 2% Goat Serum/PBS 1X, followed by secondary fluorescent antibody. Three washes with PBS were performed after each antibody’s incubation. Finally, glass slides were incubated for 20 min at RT with 1 μg/mL DAPI in PBS. After rinsing with PBS, the glass slides were mounted with coverslips using mounting media (Merck; ref. 345789).

#### SmFISH imaging

A549 cells were plated in 24-well plates on glass coverslips and treatment/infection was performed as described above. SmFISH was performed as previously described.^60^ Briefly, cells were washed twice with PBS and fixed with 4% PFA for 15 min at RT. After two PBS washes, cells were permeabilised ON with 70% ice-cold ethanol at −20°C. Cells were then equilibrated at RT and washed twice with 2x SSC buffer for 5 min each. A single wash with 20% formamide in SSC 2x was performed for 5 min at room temperature. Coverslips were then incubated with Hybridization buffer (HB - 100 mg/mL Dextran Sulfate, 20% formamide in SSC 2x), containing the smFISH probe that targets the viral spike protein in a humidified chamber overnight at 37°C in the dark.

The next day, coverslips were washed twice with 20% formamide in SSC 2x for 30 min each in the dark at RT. Nuclei were then stained 10 min with Hoechst solution at RT. Coverslips were washed twice with PBS and once with ultrapure water. Coverslips were mounted on microscope slides with mounting media and stored at 4°C at least overnight before imaging.

#### Image detection and analysis

Images were acquired, at least 2h after mounting, using an SP8 inverted confocal microscope (Leica Camera). Nuclei snaps were segmented in Fiji. Thresholding of the segmented nuclei masks was performed and watershed, then automatically counted in Fiji using the “Analyze Particles” function. The number of FAM-positive nuclei was expressed relative to the total number of nuclei within each field of view (FOV).

#### Flow cytometer-based toxicity test

Cells were seeded in 24-well plates and toxicity of Lipofectamine-transfected gapmers was determined by flow cytometry. 36 h after treatment, cells and supernatant were collected in FACS tubes, trypsinized and centrifuged at 300 g for 5 min, washed twice with PBS, and resuspended in 100 μL of Annexin-V buffer (10 mM HEPES, pH 7.4, 140 mM NaCl, 2.5 mM CaCl2 in PBS). Each tube was stained with 5 μL of APC Annexin-V (BioLegend, ref. 640920) and 5 μL of Propidium Iodide (Sigma-Aldrich; Ref: P4864). The controls included unstained and fluorescence minus one (FMO) performed on untreated and H2O2-treated samples (positive control: 20 mM H_2_O_2_ - Sigma-Aldrich; ref. 216763). The tubes were incubated for 15 min at room temperature in the dark. The samples were analyzed using a BD FACSCanto II (PI was read with a 488 nm laser and APC was read with a 633 nm laser) and normalised against the controls.

#### SHAPE-MaP

*In vivo* SHAPE-MaP was performed as previously described,^38^ with the exception that, unlike the genome-wide SHAPE-MaP analysis reported by Manfredonia et al.,^38^ the present study focused exclusively on the genomic regions selected for gapmer design (column A, Table 3). Accordingly, primer pairs were specifically designed to amplify only these target regions (Table 5). VERO-E6-TMPRSS2 cells, infected at 1.5 MOI, were treated with NAI from a 1 M stock solution (in DMSO) to a final concentration of 100 mM, while an equivalent volume of DMSO was added to control samples. For these experiments, NAI, synthetized as previously described in Spitale et al.,^63^ was used instead of the more reactive 2A3 reagent to enable direct comparison with the Manfredonia SHAPE-MaP dataset.^38^ Cells were incubated at 37°C with constant shaking for 5 min, after which the reaction was quenched by the addition of DTT to a final concentration of 0.5 M. Samples were centrifuged at 10,000 × *g* for 1 min, the supernatant was discarded, and cell pellets were resuspended in 1 mL TRIzol. Total RNA was extracted according to the manufacturer’s instructions.

Total RNA (3 μg in a final volume of 10 μL) was supplemented with 1 μL dNTP mix (10 mM each) and 1 μL of a 50 μM pooled forward primer mix (Table 5), containing the 8 forward library primers for all selected target regions. Samples were incubated at 70°C for 5 min and immediately transferred to ice for 1 min. Reverse transcription was performed in a final 20 μL reaction volume, using 200 U SuperScript II (Invitrogen; ref. 18064022), in the presence of 50 mM Tris-HCl pH 8, 75 mM KCl and 6 mM MnCl_2_, as previously described.^38^ Reactions were incubated at 42°C for 90 min, followed by 10 min at 50°C, 10 min at 55°C and 10 min at 60°C. Eight independent PCR reactions were performed for each sample, each using a specific forward and reverse primer pair corresponding to one target region (Table 5). Briefly, target regions were PCR amplified using OneTaq DNA Polymerase (NEB; Ref. M0480) and a touchdown approach, by first performing 10 cycles, starting at the optimal annealing temperature, and decreasing it by −0.5°C/cycle, followed by 20 cycles at the lowest temperature (ideal annealing temperature −5°C). The resulting amplicons were purified using NGS NucleoMag Beads (Macherey-Nagel; ref. 744970.50) at a 1:1 bead-to-sample ratio and eluted in 15 μL RNase-free water. Purified PCR products were verified on a 2% agarose gel, and each primer pair yielded a single amplicon of the expected size. The eight amplicons obtained from each sample were then pooled in equimolar amounts to a total of 50 ng DNA in a final volume of 20 μL. Libraries were prepared by blunting and dA-tailing the amplicons using the End Prep library kit (NEB; Ref: E7546) followed by NEBNext Ultra II Ligation Module kit (NEB, Ref: E7595) with the adapters obtained from NEBNext Multiplex Oligos for Illumina (NEB, Ref:E7600) following standard protocols. Reads obtained by SHAPE-MaP analysis were analyzed following the standard procedure.^38^

#### Analysis of SHAPE-MaP data

SHAPE-MaP data were analyzed using the RNA Framework suite.^64^ Briefly, reads were mapped to the SARS-CoV-2 genome reference for each specific variant (Delta B.1.617.2 (GISAID ID: EPI_ISL_3073880), SARS-CoV-2 variant Omicron BA.1 (GISAID ID: EPI_ISL_10898045) and SARS-CoV-2 Omicron subvariant BA.5 (GISAID ID: EPI_ISL_14036188)) using the *rf-map* module and Bowtie2 v2.3.5.1.^65^ Mutations were then counted using the *rf-count* module (parameters: *-m -rd*), and raw reactivities normalized using the *rf-norm* module (parameters: *-n 1000 -sm 3 -nm 3*). Normalised SHAPE reactivities are reported in File 2 (Delta), File 3 (Omicron BA.1) and File 4 (Omicron BA.5). Each file contains the nucleotide sequence together with the corresponding normalized SHAPE reactivity value for each nucleotide. Reactivity values close to 0 indicate low structural accessibility, whereas higher values indicate increasing nucleotide flexibility and accessibility. In the present study, SHAPE reactivities were classified as low (<0.4), intermediate (0.4–0.7), or high (>0.7). Positions reported as *NaN* correspond to genomic regions that were not covered by the targeted SHAPE-MaP analysis and therefore were not experimentally analyzed.

#### Software

Immunofluorescence images were processed and analyzed using Fiji. Data analysis and graph generation were performed using Microsoft Excel and GraphPad Prism 6.2. RNA structure analysis and visualization were carried out using RStudio, VARNA, and Jalview. The viral genome architecture presented in Figure 1C was created using BioRender.

### Quantification and statistical analysis

Linear mixed effects models (LMEs) were used to evaluate differences in Spike, Nucleocapsid or ISGs expression. Following this, post-hoc pairwise comparisons were conducted by first estimating marginal means, essentially, the sum of coefficients derived from the LME model. Then, the differences in marginal means between groups were divided by the standard error to calculate the t statistic and P-values were adjusted using the Tukey’s HSD method. The analysis was performed in R using lme4, lmerTest and emmeans packages. *p* > 0.05 is considered not significant (ns); *p* ≤ 0.05 is considered significantly different. Statistical significance of SHAPE-MaP reactivities was calculated using a two-tailed Pearson’s correlation coefficient. Statistical significance for the correlation between SHAPE-MaP reactivity and efficacy of the gapmers was calculated using two-way ANOVA. Statistical significance of cytokine profiles, measured by Luminex assay, of supernatants from uninfected or infected macrophages was calculated using two-sided *t* test versus the relevant control.

## Supplemental information

Document S1. Figures S1–S9

Data S1. Scripts for identification of accessible RNA regions and ASO design Scripts used to identify structurally accessible RNA regions within the SARS-CoV-2 genome and to generate the corresponding reverse-complement ASO sequences, as described in the STAR Methods.

Data S2. Normalized SHAPE-MaP reactivity values for the SARS-CoV-2 Delta variant, related to STAR Methods Per-nucleotide normalized SHAPE-MaP reactivity values for the SARS-CoV-2 Delta variant genome, generated as described in the STAR Methods.

Data S3. Normalized SHAPE-MaP reactivity values for the SARS-CoV-2 Omicron BA.1 variant, related to STAR Methods Per-nucleotide normalized SHAPE-MaP reactivity values for the SARS-CoV-2 Omicron BA.1 variant genome, generated as described in the STAR Methods.

Data S4. Normalized SHAPE-MaP reactivity values for the SARS-CoV-2 Omicron BA.5 variant, related to STAR Methods Per-nucleotide normalized SHAPE-MaP reactivity values for the SARS-CoV-2 Omicron BA.5 variant genome, generated as described in the STAR Methods.
