# Supplementary material for: Gapmers targeting conserved SARS-CoV-2 single-stranded regions reduce viral load and reshape inflammatory macrophage responses
Source: iScience. 2026 Sep 28;29(10):117639. doi: 10.1016/j.isci.2026.117639 (PMC13651819; doi:10.1016/j.isci.2026.117639)
Supplement: Supplementary file 1 [file mmc1.pdf]

iScience, Volume *29*

Supplemental information

Gapmers targeting conserved SARS-CoV-2 single-stranded regions reduce viral load and reshape inflammatory macrophage responses

Greta Gallo, Lorena Donnici, Liam S. Colley, Alessia Loffreda, Valeria Fumagalli, Matteo Iannacone, Davide Mazza, Danny Incarnato, Raffaele De Francesco, Emanuele Monteleone, Alessio De Magis, and Marco E. Bianchi

FIG. S1 Conservation of selected gapmer target regions across SARS-CoV-2 variants and SARS-CoV-1.

a) Multiple sequence alignments of the genomic regions targeted by the selected gapmers. Conserved nucleotides shared among SARS-CoV-2, SARS-CoV-1 and the analyzed SARS-CoV-2 VoCs are highlighted in blue, whereas non-conserved positions are shown in white. The candidate target regions identified from the structural analysis are highlighted in magenta, while the final gapmer target sequences selected for antisense design are highlighted in green.

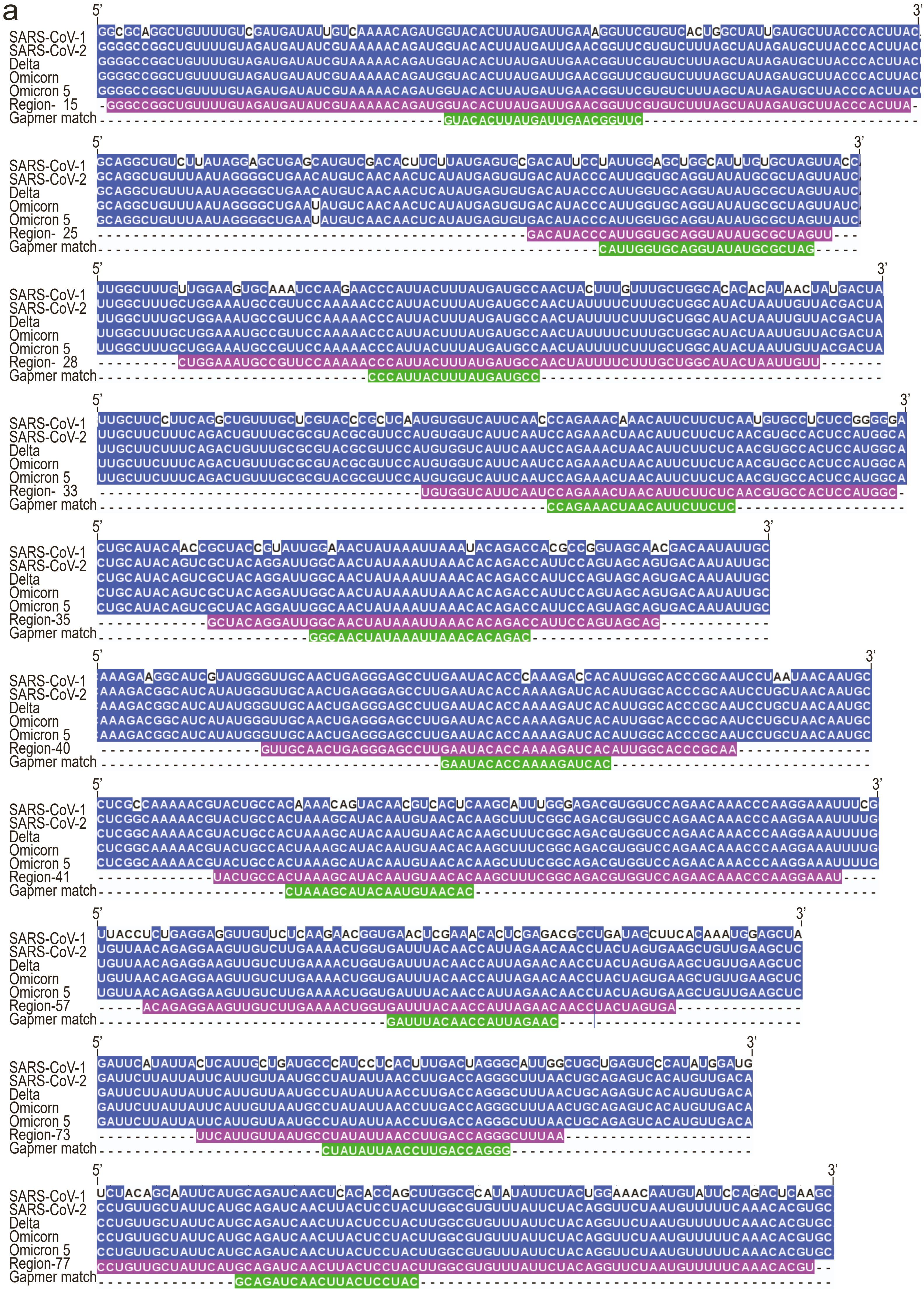
a
SARS-CoV-1
SARS-CoV-2
Delta
Omicron
Omicron 5
Region- 15
Gapmer match
GUACACUUAUGAUUGAACGGUUC
Region- 25
CAUUGGUGCAGGUAUAUGCGCUAG
Region- 28
CCCAUUACUUUAUGAUGCC
Region- 33
CCAGAAACUAACAUUCUUCUC
Region-35
GGCAACUAUAAAUUAAACACAGAC
Region-40
GAAUACACCAAAAGAUCAC
Region-41
CUAAAGCAUACAAUGUAACAC
Region-57
GAUUUACAACCAUUAGAAC
Region-73
CUAUAUUAACCUUGACCAGGG
Region-77
GCAGAUCAACUUACUCCUAC

FIG. S2 Predicted secondary structures of selected SARS-CoV-2 target regions

a) Predicted secondary structures of selected SARS-CoV-2 target regions and corresponding Gapmer-25 and 28 binding sites. The target sequences used for gapmer design are highlighted in magenta, whereas the corresponding gapmers are shown in green. The figure illustrates the structural accessibility of the selected target regions and their localization within the surrounding RNA architecture. Structures were generated using the VARNA software based on the secondary structure model reported by Manfredonia et al.[38]. Coordinates refer to nucleotide positions in the SARS-CoV-2 reference genome Leiden-0002. Within the gapmer sequences, the symbol "+" denotes LNA-modified nucleotides, whereas "*" indicates phosphorothioate linkages.

a

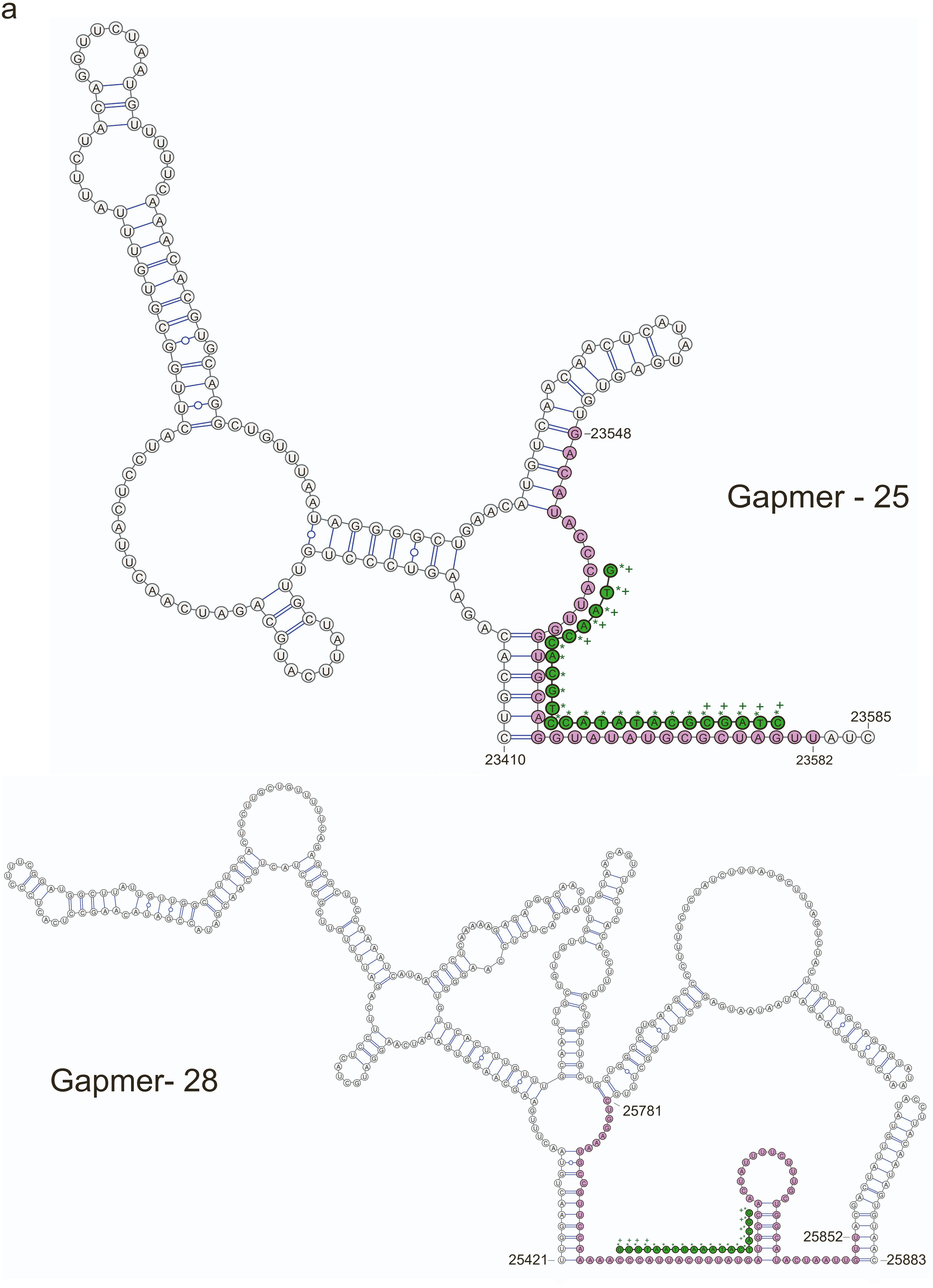
Gapmer - 25
23548
23410
23582
23585
Gapmer- 28
25781
25421
25852
25883

FIG. S3 Predicted secondary structures of selected SARS-CoV-2 target regions

a) Predicted secondary structures of selected SARS-CoV-2 target regions and corresponding Gapmer-33, 40 and 77 binding sites. The target sequences used for gapmer design are highlighted in magenta, whereas the corresponding gapmers are shown in green. The figure illustrates the structural accessibility of the selected target regions and their localization within the surrounding RNA architecture. Structures were generated using the VARNA software based on the secondary structure model reported by Manfredonia et al.[38]. Coordinates refer to nucleotide positions in the SARS-CoV-2 reference genome Leiden-0002. Within the gapmer sequences, the symbol "+" denotes LNA-modified nucleotides, whereas "*" indicates phosphorothioate linkages.

a

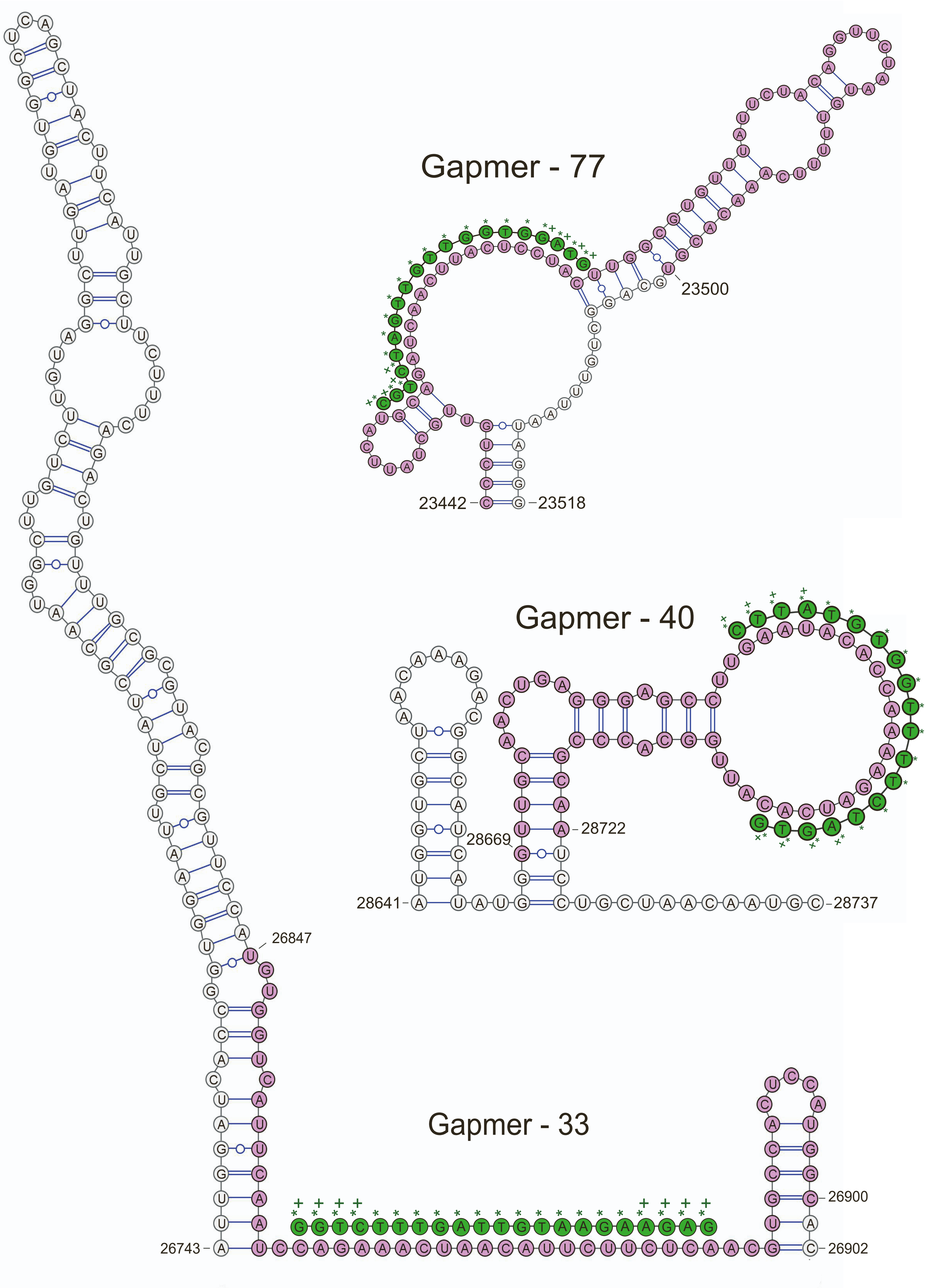
Gapmer - 77
23500
23442
23518
Gapmer - 40
28722
28669
28641
28737
26847
Gapmer - 33
26900
26743
26902

FIG. S4 Predicted secondary structures of selected SARS-CoV-2 target regions

a) Predicted secondary structures of selected SARS-CoV-2 target regions and corresponding Gapmer-73 and 15 binding sites. The target sequences used for gapmer design are highlighted in magenta, whereas the corresponding gapmers are shown in green. The figure illustrates the structural accessibility of the selected target regions and their localization within the surrounding RNA architecture. Structures were generated using the VARNA software based on the secondary structure model reported by Manfredonia et al.[38]. Coordinates refer to nucleotide positions in the SARS-CoV-2 reference genome Leiden-0002. Within the gapmer sequences, the symbol "+" denotes LNA-modified nucleotides, whereas "*" indicates phosphorothioate linkages.

a

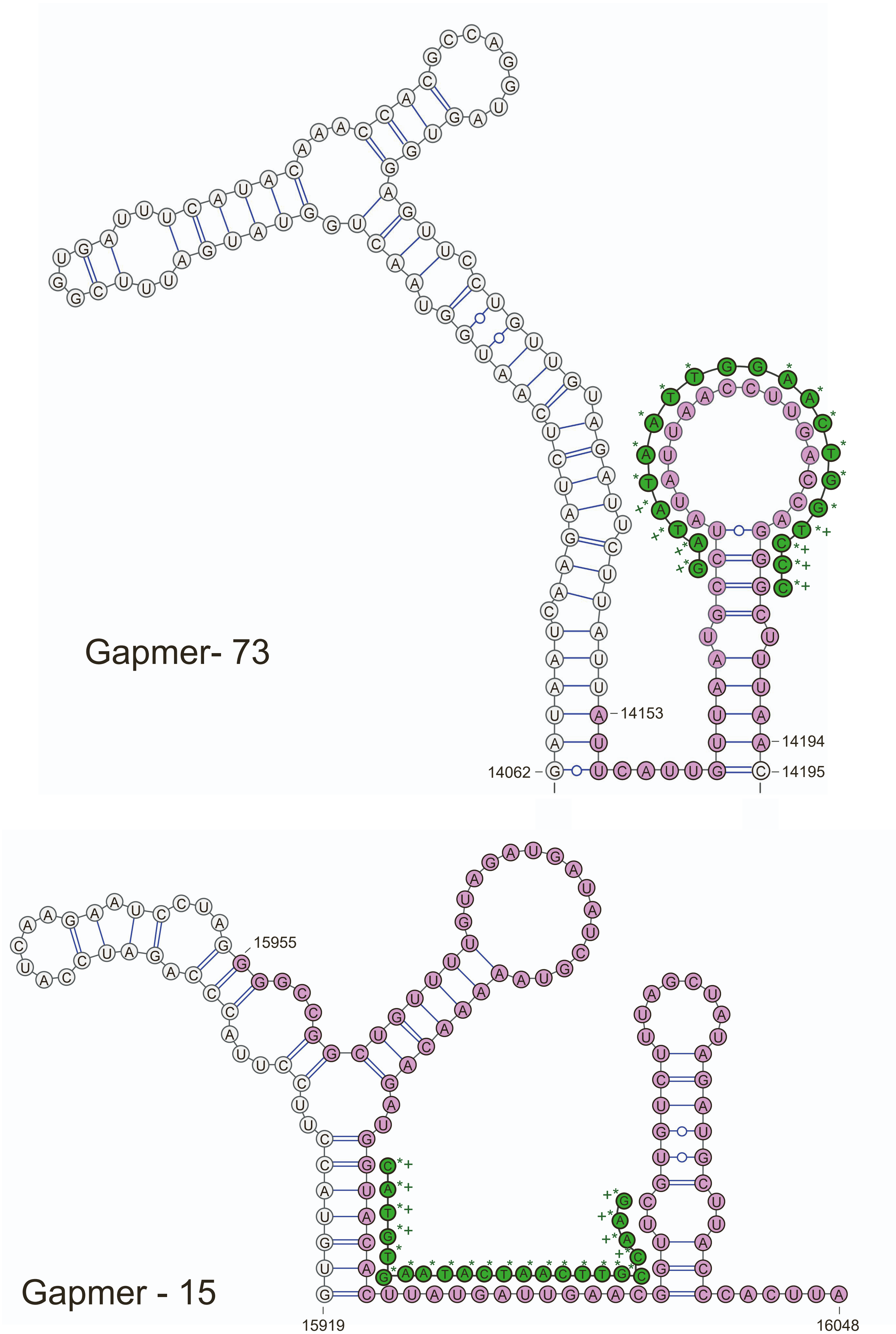

Gapmer- 73
14153
14194
14062
14195
Gapmer - 15
15955
15919
16048

FIG. S5 Efficacy of pre-ASOs in promoting cleavage of viral RNA in vitro and of corresponding gapmers in promoting destruction of genomic viral RNA in HuH-7.5 cells

a) Efficacy of pre-ASOs in promoting cleavage of viral RNA in vitro. qPCR on ASO target regions performed in vitro on cDNA synthesized from viral RNA treated with two different concentrations (5 and 10 μM) of pre-ASOs and RNaseH. Each gapmer was tested in biological triplicates and normalized to UT. Bars represent mean ± SD of ΔΔCt values relative to UT. Heatmaps below the graphs show relative expression levels of each target compared to UT. b) Efficacy of gapmers in promoting destruction of genomic viral RNA in HuH-7.5 cells. Left panel: qPCR on the S genomic region, performed on cDNA synthesized from intracellular RNA isolated from infected HuH-7.5 cells. Right panel: qPCR targeting the Nucleocapsid (N1) genomic region, performed on extracellular RNA recovered from the same experiment. Each gapmer was tested in technical triplicates from biological duplicates and normalized to untreated (UT) control. Bars represent mean ± SD of ΔΔCt values relative to UT. Heatmaps below the graphs show relative expression levels of each target compared to UT. Statistical significance is calculated using LMEs and post-hoc pairwise comparison, and is indicated as follows: is indicated as follows: ns (not significant), $*p \leq 0.05$, $**p \leq 10^{-3}$, $***p \leq 10^{-10}$.

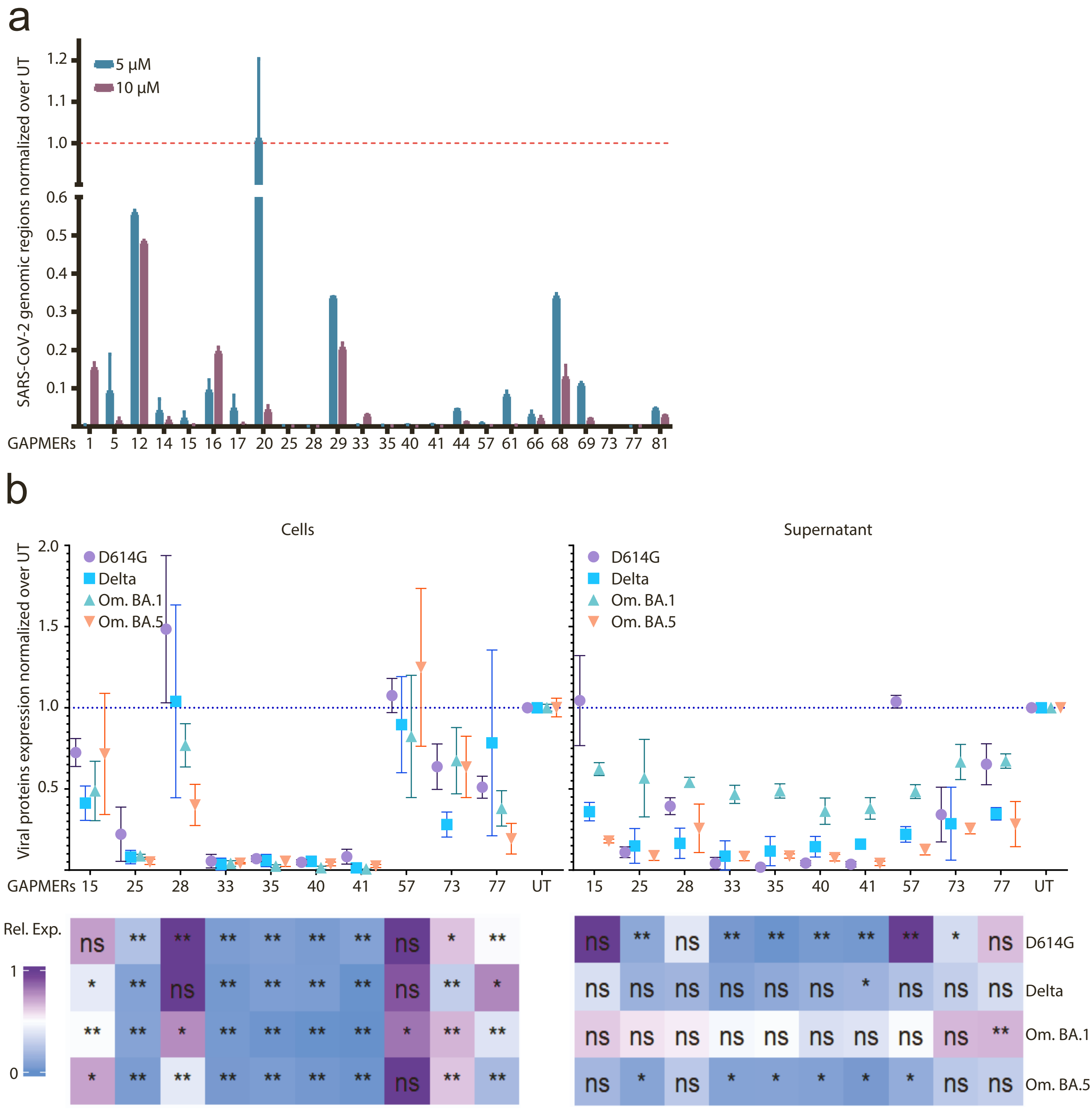
a
SARS-CoV-2 genomic regions normalized over UT
5 μM
10 μM
1.2
1.1
1.0
0.6
0.5
0.4
0.3
0.2
0.1
GAPMERs 1 5 12 14 15 16 17 20 25 28 29 33 35 40 41 44 57 61 66 68 69 73 77 81
b
Cells
Supernatant
Viral proteins expression normalized over UT
D614G
Delta
Om. BA.1
Om. BA.5
2.0
1.5
1.0
0.5
GAPMERs 15 25 28 33 35 40 41 57 73 77 UT
15 25 28 33 35 40 41 57 73 77 UT
Rel. Exp.
1
0
D614G
Delta
Om. BA.1
Om. BA.5

FIG. S6 Imaging of viral RNA in treated and untreated cells

Representative microscopy images from A549-hACE2 cells. Cells were lipofectamine-transfected with the indicated gapmers and infected with SARS-CoV-2 variants. The smFISH was performed using a probe targeting the SARS-CoV-2 S protein (red); nuclei were stained with Hoechst (blue). The images belong to the same experiments shown in Fig. 3a. Scale bar, 50 µm.

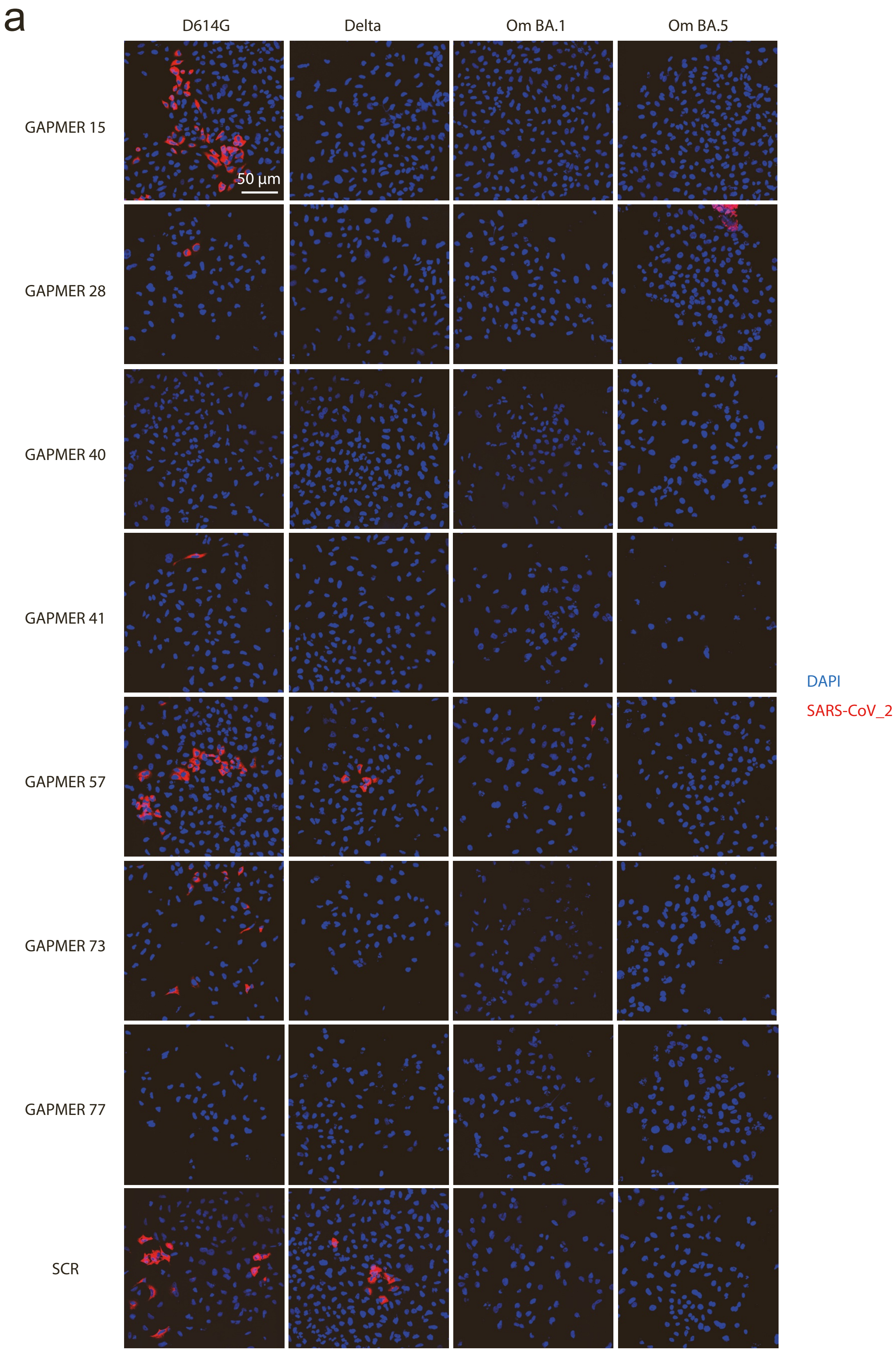
a
D614G
Delta
Om BA.1
Om BA.5
GAPMER 15
GAPMER 28
GAPMER 40
GAPMER 41
GAPMER 57
GAPMER 73
GAPMER 77
SCR
50 µm
DAPI
SARS-CoV_2

FIG. S7 Viability assay performed by flow cytometry on A549-hACE2 cells transfected with gapmers using Lipofectamine.

The experiment was performed in biological triplicates. The graphs show a representative sample for each gapmer tested. Quadrant Q1 (bottom right) represents apoptotic cells (annexin V +, PI -), quadrants Q2–Q3 (top right and left) represent dead cells (PI +), and quadrant Q4 (bottom left) contains live cells (annexin V -, PI -), each labelled with its corresponding percentage.

a

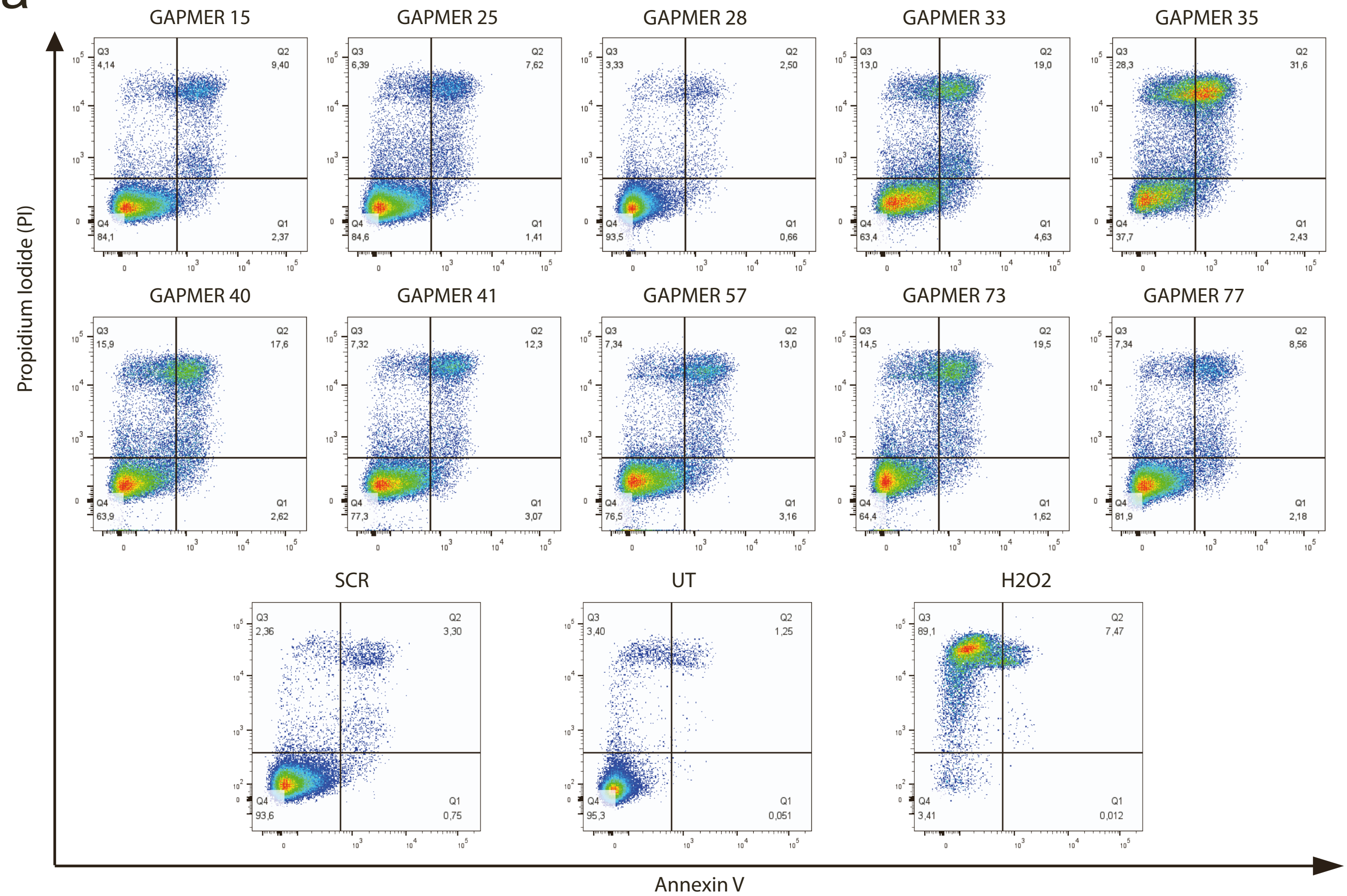

GAPMER 15
GAPMER 25
GAPMER 28
GAPMER 33
GAPMER 35
GAPMER 40
GAPMER 41
GAPMER 57
GAPMER 73
GAPMER 77
SCR
UT
H2O2
Propidium Iodide (PI)
Annexin V
Q1
Q2
Q3
Q4

FIG. S8 Gapmers significantly moderate interferon responses in infected cells

a) qPCR of ISGs, performed on cDNA synthesized from intracellular RNA isolated from A549-hACE2 cells infected with variants Omicron 1(left panel) and 5 (right panel). Bar graphs represent the mean ± SD of ΔΔCt values. All experiments were performed in technical triplicates of biological duplicates. Heatmaps below the graphs show relative expression levels of each target compared to control. Statistical significance is calculated using LMEs and post-hoc pairwise comparison, and is indicated as follows: ns, not significant, $^{*}p \leq 0.05$, $^{**}p \leq 10^{-3}$, $^{***}p \leq 10^{-10}$.

a

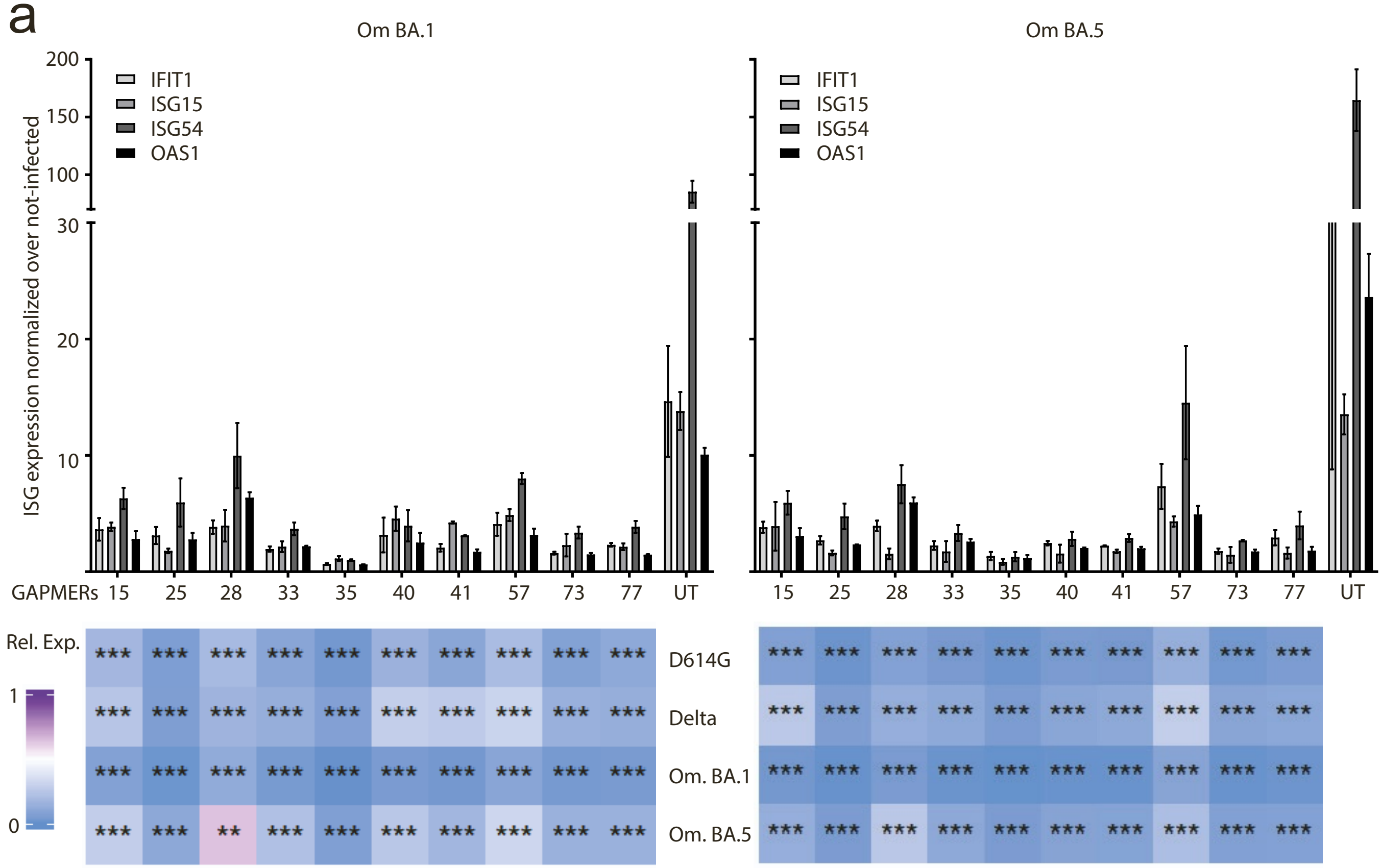
Om BA.1
Om BA.5
IFIT1
ISG15
ISG54
OAS1
ISG expression normalized over not-infected
200
150
100
30
20
10
GAPMERs
15
25
28
33
35
40
41
57
73
77
UT
Rel. Exp.
1
0
D614G
Delta
Om. BA.1
Om. BA.5
***
**

FIG. S9 Cytokine profiles in infected monocyte-derived human macrophages

a) Cytokine profiles of supernatants from not infected macrophages (grey), at 24 h. p.i. (orange) and 48 h p.i. (red). b) Cytokine profiles of supernatant from macrophages at 48 h; p.i. untreated (red), Gapmer-35 treated (green) and scramble treated (grey). A-b) Bar graphs represent the mean ± SD of ΔΔCt values. Statistical significance is calculated using two-sided T-test versus the relevant control (grey bar) and indicated as follows: ns, not significant, *$p \leq 0.05$, **$p \leq 0.01$, ***$p \leq 10^{-3}$, ****$p \leq 10^{-4}$.

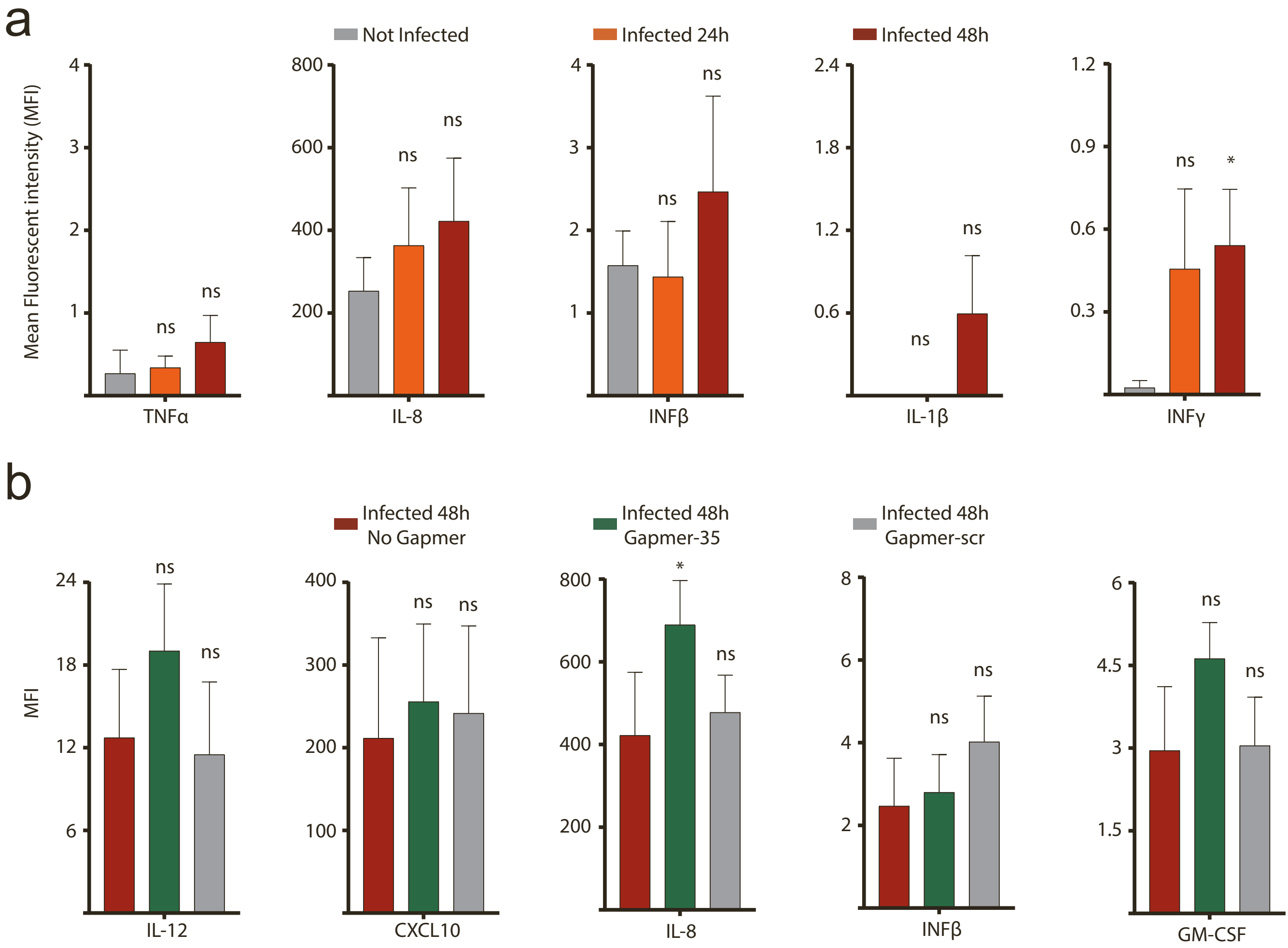
a
Not Infected
Infected 24h
Infected 48h
Mean Fluorescent intensity (MFI)
TNFα
IL-8
INFβ
IL-1β
INFγ
ns
*
b
Infected 48h No Gapmer
Infected 48h Gapmer-35
Infected 48h Gapmer-scr
MFI
IL-12
CXCL10
IL-8
INFβ
GM-CSF
